# Tumor-associated macrophages: an effective player of the tumor microenvironment

**DOI:** 10.3389/fimmu.2023.1295257

**Published:** 2023-11-16

**Authors:** Udit Basak, Tania Sarkar, Sumon Mukherjee, Sourio Chakraborty, Apratim Dutta, Saikat Dutta, Debadatta Nayak, Subhash Kaushik, Tanya Das, Gaurisankar Sa

**Affiliations:** ^1^ Division of Molecular Medicine, Bose Institute, Kolkata, India; ^2^ Central Council for Research in Homeopathy (CCRH), New Delhi, India

**Keywords:** tumor-associated macrophages, pro-tumor immunity, cancer stem cells, tumor microenvironment, anticancer therapy, immunotherapy, immunosuppression, macrophage reprogramming

## Abstract

Cancer progression is primarily caused by interactions between transformed cells and the components of the tumor microenvironment (TME). TAMs (tumor-associated macrophages) make up the majority of the invading immune components, which are further categorized as anti-tumor M1 and pro-tumor M2 subtypes. While M1 is known to have anti-cancer properties, M2 is recognized to extend a protective role to the tumor. As a result, the tumor manipulates the TME in such a way that it induces macrophage infiltration and M1 to M2 switching bias to secure its survival. This M2-TAM bias in the TME promotes cancer cell proliferation, neoangiogenesis, lymphangiogenesis, epithelial-to-mesenchymal transition, matrix remodeling for metastatic support, and TME manipulation to an immunosuppressive state. TAMs additionally promote the emergence of cancer stem cells (CSCs), which are known for their ability to originate, metastasize, and relapse into tumors. CSCs also help M2-TAM by revealing immune escape and survival strategies during the initiation and relapse phases. This review describes the reasons for immunotherapy failure and, thereby, devises better strategies to impair the tumor–TAM crosstalk. This study will shed light on the understudied TAM-mediated tumor progression and address the much-needed holistic approach to anti-cancer therapy, which encompasses targeting cancer cells, CSCs, and TAMs all at the same time.

## 1 Introduction

Cancer is one of the highest causes of morbidity globally (1). Accumulation of genetic mutations brings about this disease; however, the progression would not have been possible by itself alone, but rather with the help of a multitude of factors constituted in the tumor microenvironment (TME). The heterogeneous cellular and non-cellular elements of the TME, such as immune cells, cancer-associated fibroblasts (CAFs), matrix components, and chemokines, operate either against or with the disease (2). To combat transformed cells, known as the elimination phase, inflammation in the TME later turns to an equilibrium phase, with the coexistence of surviving cancer cells along with inflammatory subsets (3). Eventually, chronic inflammation ends up helping the tumor (escape phase) (3), pointing towards the two hallmarks of cancer, namely, tumor-promoting inflammation and avoiding immune destruction (4). Cancer, being a master manipulator, evades the anti-tumor immune surveillance via escaping detection, inducing death, or changing it to a phenotype that serves its purpose (5, 6).

Tumor-associated macrophages (TAMs) occupying more than 50% of tumor-infiltrating cells can be broadly classified as M1-like, pro-inflammatory subtype and M2-like, immunosuppressive subtype (3, 7). During the early stages of cancer detection by immune cells, tissue-resident macrophages (TRMs) attack them by developing pro-inflammatory conditions and presenting phagocytosed antigens on their major histocompatibility complex (MHC) class II receptor (8, 9).

As a result, an adaptive immune response is generated with the invasion of anti-tumor M1 macrophages, CD8^+^ T cells, and natural killer (NK) cells, which maintains the inflammatory conditions to challenge cancer cells while also constantly replenishing their population (9, 10). Because cancer has an immunomodulatory feature, this neoplastic fraction develops ways to prevent phagocytosis, elude detection, recruit pro-tumor M2 macrophages, and, most intriguingly, produce cytokines to shift the M1 to the M2 type (11). As a result, what started as an effective tumor-eliminating response ends up with a tumor-promoting one, exacerbating the disease. When discussing tumor elimination, it is appropriate to emphasize the tiny subpopulation known as cancer stem cells (CSCs), which, according to mounting evidence, is the “root cause” of therapeutic failures, relapse, tumor progression, invasion, metastasis, and even cancer initiation (12, 13). CSCs employ diverse modalities and hide within the cancer niche to protect themselves from stress signals such as chemotherapeutic drugs and immune attacks (12). Understanding the CSC–TAM crosstalk is therefore critical to get the big picture.

In this review, we will address the important role that TAMs play in TME, whether to suppress or aid cancer. We will have a better understanding of the many underlying processes of the bidirectional interaction between cancer and TAMs, as well as that of CSC–TAM, which not only shapes the tumor landscape but also defines the immunological environment, as well as abetting disease progression and therapy failure. Understanding this crosstalk would aid us in developing strategies to effectively target cancer with its supporting immune arm.

## 2 Tumor microenvironment

The tumor body is not just a collection of cancer cells but also contains a variety of resident and infiltrating host cells (14). Tumor cells constantly compel the TME to undergo extensive molecular, cellular, and physical alterations to sustain their growth and development. The composition of a developing TME is a complex, dynamic entity, which varies according to the stages and kind of tumor with distinctive features including immune cells, stromal cells, blood vessels, and extracellular matrix (ECM). Although various adaptive and innate immune cells that promote or inhibit tumor growth infiltrate the tumor site, it is shown that TME actively fosters cancer development rather than simply acting as a silent observer (15). Tumor growth and its constantly accumulating mutations change the TME immunophenotype from immunogenic to tolerogenic, reprogramming pro-inflammatory immune cells to behave in favor of tumor growth, provide resistance to applied therapies, and fail to perform their intended function (10).

**Figure 1:**
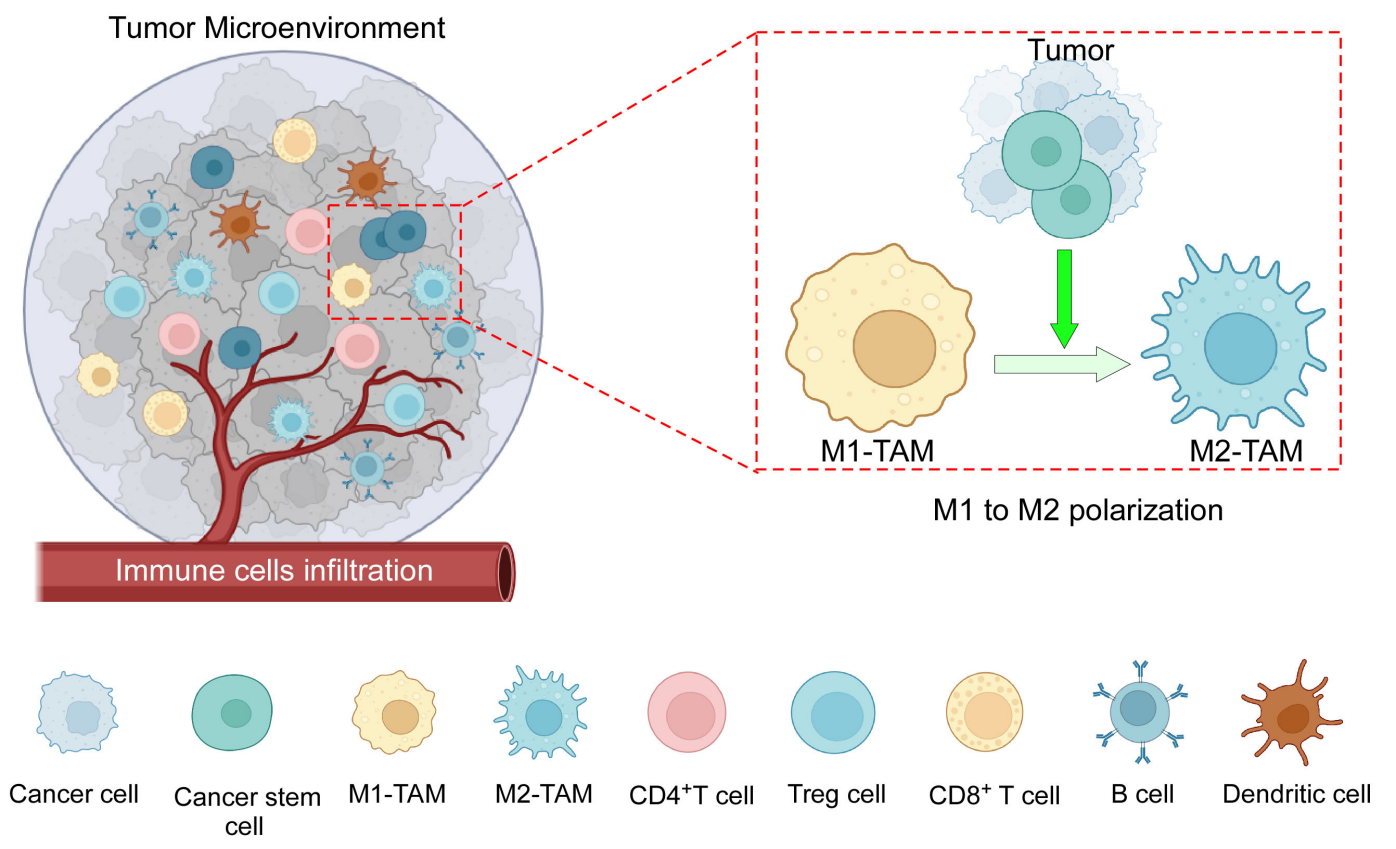
The components of the TME: TME consists of an intricate interplay among various cellular components, including tumor cells, CSCs, tumor-associated macrophages (M1 and M2), CD4^+^ and CD8^+^ T cells, B cells, dendritic cells, immune-suppressive Treg cells, as well as other cells, and a complex network of dysregulated vasculature. In addition to several mechanisms, cancer cells along with CSCs play an active role in the polarization of pro-inflammatory M1 macrophages towards anti-inflammatory M2 macrophages. Created with BioRender.com.

TME immune components include both pro- and anti-inflammatory cells of innate and adaptive immunity (**Figure 1**). Macrophages are particularly significant because they influence both innate and adaptive immune responses (16). TAMs in tissues were assumed to originate solely from bone marrow (BM)-derived monocytes. Recent evidence suggests the existence of TRMs, which evolve from embryonic progenitors and can survive without the assistance of BM-derived monocytes (17, 18). It is recognized that during the early stages of cancer, only embryonic lineage-derived TRMs aid tumor progression, and that eventually, in response to signals from developing tumors, blood-derived monocytes infiltrate and further enhance the process (17). In the murine mammary carcinoma model, TRMs decrease in number whereas BM-derived monocytes increase with the advancement of cancer (19). However, in tumors such as pancreatic ductal adenocarcinoma, both types of TAMs are present at the same time, with TRMs secreting significant levels of fibrotic proteins and infiltrating macrophages primarily serving as antigen presenters (18). Furthermore, TRMs in ovarian cancer are thought to induce metastasis and maintain the CSC population (20). TRMs are also found to be the most common type of macrophage associated with brain tumors (21), and they have been linked to glioma formation and progression (22). In TME, when considering the overall TAM population, they can be broadly subtyped as anti-tumor M1 and pro-tumor M2 phenotypes. Anti-tumor M1 macrophage demonstrates tumoricidal activity, whereas immunosuppressive M2-polarized macrophages, commonly termed as M2-TAMs, are the most prominent immune cell population within the TME (9). Incidentally, infiltrating macrophages in the majority of tumors are also predominantly of the pro-tumor M2 phenotype. TAMs have a range of roles depending on tumor type, but they are generally important in all stages of carcinogenesis, from tumor initiation to secondary tumor progression. They contribute to angiogenesis, lymphangiogenesis, immunological suppression, hypoxia-induced tumorigenesis, metastasis, and drug resistance, among others (7). TAMs and CSCs have recently been discovered to communicate with each other (23). TAMs are emerging as a critical diagnostic indicator due to the frequent association between their abundance and a poor prognosis (24). As a result, it is critical to investigate TME-induced macrophage skewing and TAM-related pro-tumorigenic effects.

## 3 Types of macrophages

Multiple macrophage phenotypes have been identified so far based on diverse surface receptor expressions, secretory patterns, and activities (25). The multiple unique markers that macrophages express on their membranes result in a range of phenotypes dependent on TME signals, leading to a high degree of plasticity. While TAMs are closely associated with the tumor, cancer-associated macrophage-like (CAML) cells are disseminated tumor cells found in peripheral blood (26). While differentiated macrophages are a rare phenomenon as a circulating population of cells, CAML is defined as macrophages with phagocytosed tumor fraction, has been studied in breast, pancreatic, and prostate cancer, and may serve as a biomarker for these cancers (27). CAMLs are more commonly found in people with advanced malignancies than in those with benign tumors. Surprisingly, the number of CAMLs increases following chemotherapy, which may be related to the efficiency of the treatment because increased phagocytosing macrophages are proportionate to dying cancer cells (26, 27). Augustyn et al., on the other hand, used data from a phase 2 clinical trial (NCT02525757) to show that the existence of giant CAMLs was associated with metastases and poor survival despite immunotherapy and chemoradiation (28). Furthermore, patients with programmed cell death ligand 1 (PDL1) expressing CAML when treated with immunological checkpoint inhibitors (ICIs) had significantly greater overall survival than those who were not treated with ICIs in metastatic lung cancer (29). In general, TAMs are classified as either traditionally activated anti-tumor M1 or alternatively activated pro-tumor M2 phenotypes. According to *in vivo* wound healing studies, the early stages of wound healing are characterized by the activation of pro-inflammatory M1-like macrophages, which gradually give way to an anti-inflammatory M2-like macrophage phenotype (30). The macrophage colony-stimulating factor-1 receptor (CSF1R) is the primary lineage regulator of virtually all macrophages, regardless of origin. This class III transmembrane tyrosine kinase receptor is expressed by the majority of mononuclear phagocytic cells (25). IFNγ and IL1β, secreted by Th1 cells and bacterial lipopolysaccharide (LPS), drive macrophages to polarize towards the M1 phenotype, whereas IL4 and IL13, secreted by Th2 cells, cause the M2 phenotype to be dichotomized (31).

Macrophages in the M1 end of the continuum have a pro-inflammatory phenotype and express the surface markers CD86 and CD64 (32); MHC-II and macrophage receptor with collagenous structure (MARCO) (33, 34); nitric oxide synthase-2 (NOS2) and suppressor of cytokine signaling-1 (SOCS1); and pro-inflammatory cytokines (IL6, IL12, IL1β, and TNFα) and chemokine ligands (CCL2, CCL5, CXCL9, CXCL10, and CXCL11) (32). All of these indicators show their strong phagocytic and cytotoxic power, ability to draw T and B cells to the infection site, and prodigious ability to deliver antigens (35). In contrast, pro-tumor M2 macrophages with surface markers CD36, CD206, and CD163 are immunosuppressive and anti-inflammatory, helping with tissue repair, angiogenesis, and phagocytosis to reduce and “clean up” after inflammation. They are also Th2 activators and Th1 inhibitors (25, 35–37). M2-like macrophages are typically characterized by their poor ability to present antigens, having low IL12 and high IL10, IL4, and IL13 secretory profiles (35). M2 macrophages also express/secrete transforming growth factor-beta (TGFβ), peroxisome proliferator-activated receptor-gamma (PPARγ), CCL14, CCL22, and arginase-1 (ARG-1) (38).

M2-like macrophages are more functionally diverse than M1-like macrophages because they have many subtypes (M2a, M2b, M2c, and M2d), each with a distinct combination of cytokine and chemokine profiles (39). M2a macrophages express higher levels of IL10, TGFβ, and the chemokines CCL17, CCL18, CCL22, and CCL24, all of which are linked to Th2-polarized allergic inflammation. IL4 and/or IL13 stimulate the production of M2a macrophages. Immune complexes (ICs), LPS, Toll-like receptors (TLRs), or the IL1 receptor antagonist (IL1ra), on the other hand, sustains M2b macrophages, which are characterized by the production of TNFα, IL1β, IL6, IL10, and CCL1. A TGFβ-, glucocorticoid (GC)-, prostaglandin E2-, and IL10-rich environment induces M2c macrophages, which continue to express IL10 and TGFβ; thus, they are crucial regulators for inflammation resolution and tissue healing. Finally, M2d macrophages have been shown to contribute to angiogenesis by expressing vascular endothelial growth factor (VEGF) and IL10 when stimulated by TLR, adenosine A2A receptor ligands, and IL6 (40, 41).

In the context of cancer, these pro-tumor M2 macrophage subsets share the function of tumor development and immune response suppression via multiple pathways (42). For example, VEGF and CCL18, which are released by M2a macrophages, promote breast cancer cell motility and angiogenesis (43, 44). Furthermore, M2a macrophages contribute to the development of lung cancer cells via the IL4/STAT6 signaling pathway. STAT6-expressing M2a macrophages are required for tumor cell growth (45). IL4, which is released by both tumor cells and M2a macrophages, promotes more macrophages to polarize to the M2a phenotype, resulting in a positive feedback loop (45). M2b macrophages proliferate and replace M1 macrophages as hepatocellular carcinoma (HCC) advances (46). These cells release CCL1 in order to attract Th2 and Treg cells that express CCR8, thereby promoting a pro-tumorigenic environment (47). The CCL1/CCR8 signaling mechanism also enhances tumor cell motility, proliferation, and metastasis (48, 49). Furthermore, M2b macrophages express higher levels of indoleamine 2,3-dioxygenase (IDO), IL10, and IL6, all of which are immunosuppressive factors (50). M2b macrophage-secreted IL10 promotes Treg cell differentiation from naive T cells (42), whereas secreted IL6 activates Th2 cells, which promote tumor progression (51). In breast cancer patients, the percentage of circulating M2c macrophages is associated with a poor prognosis (52). Yuan et al. demonstrated that M2c macrophages promote lung tumor growth (53). Kim et al. (54) revealed evidence that IL10-induced M2c macrophages promote tumor development in mouse melanoma models. Furthermore, both *in vitro* and *in vivo*, M2c macrophages enhanced endothelial cell mobility and tube formation, implying that M2c may boost tumor growth through increased angiogenesis (55, 56). M2d macrophages in gastric cancer release a number of pro-tumorigenic molecules, including IL10 and TGFβ, to promote cancer cell proliferation and migration (57). M2d macrophages release VEGF and matrix metalloproteinase 9 (MMP9), which promote ECM breakdown, angiogenesis, and metastasis (58). They also produce IL6 in various cancer. The canonical IL6/JAK/STAT3 pathway is related to survival, angiogenesis, metastasis, proliferation, and drug resistance. M2d macrophages also express immunosuppressive factors such as IDO, IL10, and PDL1 (42, 58).

## 4 Macrophage polarization states in the TME

M1-like macrophages penetrate the TME during the early phase of tumor by antigen presentation and secreting CXCL9, CXCL10, and CXCL11, which chemoattract CXCR3-expressing effector immune cells like CD8^+^ cytotoxic T cells and NK cells (9, 59). A pro-inflammatory environment containing IFNγ is associated with the preservation of CXCL10^+^ M1 macrophages (60, 61). In M1 macrophages, IFNγ activates the STAT1 pathway, which is responsible for the production of CXCL10 (62–64). In ovarian cancer, CXCL10^+^IRF1^+^STAT1^+^ M1 resulted in improved antigen processing capacity and T-cell infiltration, as well as a better patient prognosis (60). Effector and memory T cells unlike naïve T cells express CXCR3 (65). As a result of increased infiltration of effector cells, elevated CXCL10 expression has been associated with patient survival in ovarian cancer (66). CXCR3 inhibition reduced CD8^+^ T-cell infiltration and accelerated tumor progression in mouse models (67). CXCR3 is shown to be expressed in Tregs in HCC, which can hinder effector immune cells from acting due to competitive recruitment in the TME (68). Furthermore, CXCL10 induces chemotaxis of CXCR3^+^ NK cells, resulting in tumor regression (69). STAT3 activation in CD8^+^ T cells suppresses IFNγ production, which, in turn, suppresses CXCL10 release from TAMs. STAT3 inhibition resulted in an increase in CXCR3-expressing CD8^+^ T cells and a better prognosis (70).

Trafficking of effector T cells and NK cells affected the release of pro-inflammatory cytokines (IFNγ, GMCSF, and TNFα) and chemokines (CCL4, CCL5, and CCL23) in the TME, assisting in the recruitment of extra effector immune cells and signaling of anti-tumorigenic pathways (71). For efficient tumor killing, the anti-tumor M1 macrophage expresses IL12, IL1, and iNOS (72). In several cancers, an elevated M1/M2 TAM ratio is associated with longer survival and better clinical outcomes, including small cell lung cancer (73), non-small cell lung cancer (74), colorectal cancer (75), ovarian cancer (72), breast cancer (76), oral squamous cell carcinoma (77), and others (78). LPS-induced TLR4 alone or in combination with IFNγ activates the PI3K-mTOR-AKT (phosphoinositide-3-kinase-mammalian target of rapamycin) pathway, which leads to the polarization of anti-tumor M1 macrophages and the suppression of cancer cell proliferation (79). Furthermore, phosphatase and tensin homolog (PTEN) regulates the inflammatory response via M1 polarization (80). NFκB (81) is a major regulator of M1 activation. TNFα is a positive regulator of M1 polarization and a negative regulator of M2 polarization when the NFκB pathway is activated. Furthermore, the myeloid differentiation primary response 88 (MyD88) suppresses M2 gene expression in TAMs, resulting in an anti-tumor M1 phenotype (82).

### 4.1 M1 macrophages in TME

M1-like macrophages perform their anti-cancer function by first efficiently distinguishing cancer cells from surrounding healthy cells by recognizing altered or cancer-specific antigens. Macrophages recognize altered carbohydrate structures (or glycosylation) that cancer cells occasionally present on their cell surfaces. Some tumor antigens, such as carcinoembryonic antigen and Tn antigen, are glycosylated molecules that lectin-like receptors on macrophage cell membranes recognize (83). Secondly, M1 eventually kills cancer cells via directly inducing cytotoxicity, phagocytosis, and antibody-dependent cell-mediated cytotoxicity (ADCC) (84).

The first step is somewhat slow, lasting 1 to 3 days and involving several stages such as the formation of ROS (reactive oxygen species) and RNS (reactive nitrogen species), as well as the production of IL1 and TNF to destroy cancer cells (85). Activated anti-tumor M1 macrophages target tumor cells by generating ROS and nitric oxide (NO), causing DNA damage, cytotoxicity, and apoptosis (86). M1 macrophages sustain themselves as well as induce NK cell and cytotoxic T-cell infiltration and activation in the tumor site by secreting substantial amounts of pro-inflammatory cytokines IFNγ and IL12 with anti-tumor activity, indicating an indirect mechanism of inhibiting cancer progression (87). Recognizing cancer cells and effectively phagocytosing them, on the other hand, suggests their ability as innate immune effectors, which later cross-primes adaptive immune response by presenting antigens on their surface (88). M1 uses ADCC as an adaptive response, employing anti-tumor antibodies for opsonization, which is a quicker process (89). After adhering to the Fc region of the antibodies, macrophages can phagocytose cancer cells coated with antibodies. To avoid M1-mediated elimination, tumor cells downregulate recognition molecules and activate counter-signals, such as PD1 activation in TAMs (88).

Anti-tumor M1 macrophages also stimulate the immune system’s anti-tumor activity, slowing cancer growth. Dectin1 expression on M1 macrophages increases NK cell-mediated tumor cell death (49). M1-like macrophages increase the number of both total and activated NK cells in the fibrotic liver, resulting in TRAIL-induced cell death (90). M1 also has increased co-stimulatory activity for effector T-cell activation (87). Other mechanisms include decreased VEGF, MMP, and CCL18 secretion relative to pro-tumor M2 macrophages, which prevents angiogenesis and metastasis (87). The key treatment options to target TAMs in the TME include TAM depletion, re-polarization into a more pro-inflammatory (M1) state, or reawakening of M1 macrophages’ anti-cancer potential (16).

### 4.2 TME shifts anti-tumor M1 to pro-tumor M2 phenotype

As the tumor grows bigger, TME alters the ratio from M1 to M2 (71) (**Figure 1**). Malignant cells release M2-chemoattracting cytokines such as IL10, CCL2/CCL3/CCL4/CCL5/CCL7/CCL8, CXCL12, VEGF, platelet-derived growth factor (PDGF), and CSF1 to attract monocytes and M0 macrophages to the TME and differentiate them into M2 phenotype (72). TRMs are the first to be impacted by the pro-tumor TME machinery, resulting in an M2-rich tumor mass, which is frequently associated with a poor prognosis in numerous malignancies (91). Exosomes generated by HCC remodeled macrophages to the M2 subtype, causing pro-tumor responses in other immune compartments (92).

Interestingly, macrophage polarization in the TME is also correlated with distinct metabolic characteristics of glucose, lipid, and glutamine metabolism (93). Such metabolic alterations can determine the phenotype and functionality of TAMs in promoting cancer progression. Cancer cells utilize their metabolic by-products to alter the phenotype and functional activity of tumor-infiltrating immune cells for their growth. Lactic acid produced by the “Warburg effect” shifts the macrophages toward a more M2-like state (94). In ovarian cancer cell membranes, cholesterol efflux drives TAM reprogramming and tumor progression by inducing an IL4-mediated Th2-like environment (95). In addition, glutamate-ammonia ligase (GLUL) favors M2-like TAM polarization by catalyzing the conversion of glutamate into glutamine, whereas GLUL inhibition reverses the change (96). The recruitment and induction of macrophages in the TME can be significantly influenced by hypoxia. TAMs are drawn to and found in greater numbers in hypoxic areas of TME as a result of HIF1/2 and endothelin-2, released by hypoxic cancer cells (97). Damage-associated molecular pattern (DAMP), which is released as a consequence of hypoxia, induces the number of M2-TAM in the TME, which, in turn, secretes high levels of pro-angiogenic factor VEGF (98).

Other immune cells such as T-regulatory (Treg) cells, myeloid-derived suppressor cells (MDSCs), and B cells also control the polarization of macrophages. TAMs’ metabolic adaptability, mitochondrial integrity, and survival rate are indirectly, yet specifically maintained by Treg cells (99). In addition, MDSCs also regulate TAM differentiation in the TME (100). B cells can induce M2b macrophage polarization in human HCC, as well as suppress the function of M1 macrophages in the TME to promote the proliferation of cancer cells (101).

The polarization state of macrophages is a continuum and relates to the activation state of a macrophage at a specific moment, depending on the availability of numerous signals from other components of the TME (102). The M1 phenotype can transform into an M2 phenotype or *vice versa* in response to TME factors such as the accessibility of cytokines, growth factors, hypoxia, and the influence of other immune cells. There is more to macrophage divergence than the binary designation of M1 and M2, which represents the extremes on this spectrum and many intermediate cells may co-express both the markers of M1 and M2, indicating the complexity of polarization (103).

## 5 Pro-tumor M2-TAMs promote various aspects of tumor growth

### 5.1 TAMs encourage increased tumor cell proliferation

Replicative immortality being a crucial hallmark of cancer is enabled with increased infiltration of TAM in the case of many human malignancies such as breast cancer, endometrial cancer, and renal cell cancer (104). *In vivo* co-culture studies of macrophage and tumor cells highlighted the importance of infiltrated TAMs for the growth of tumor cells, and resultantly, depletion of TAMs effected the opposite (105).

**Figure 2:**
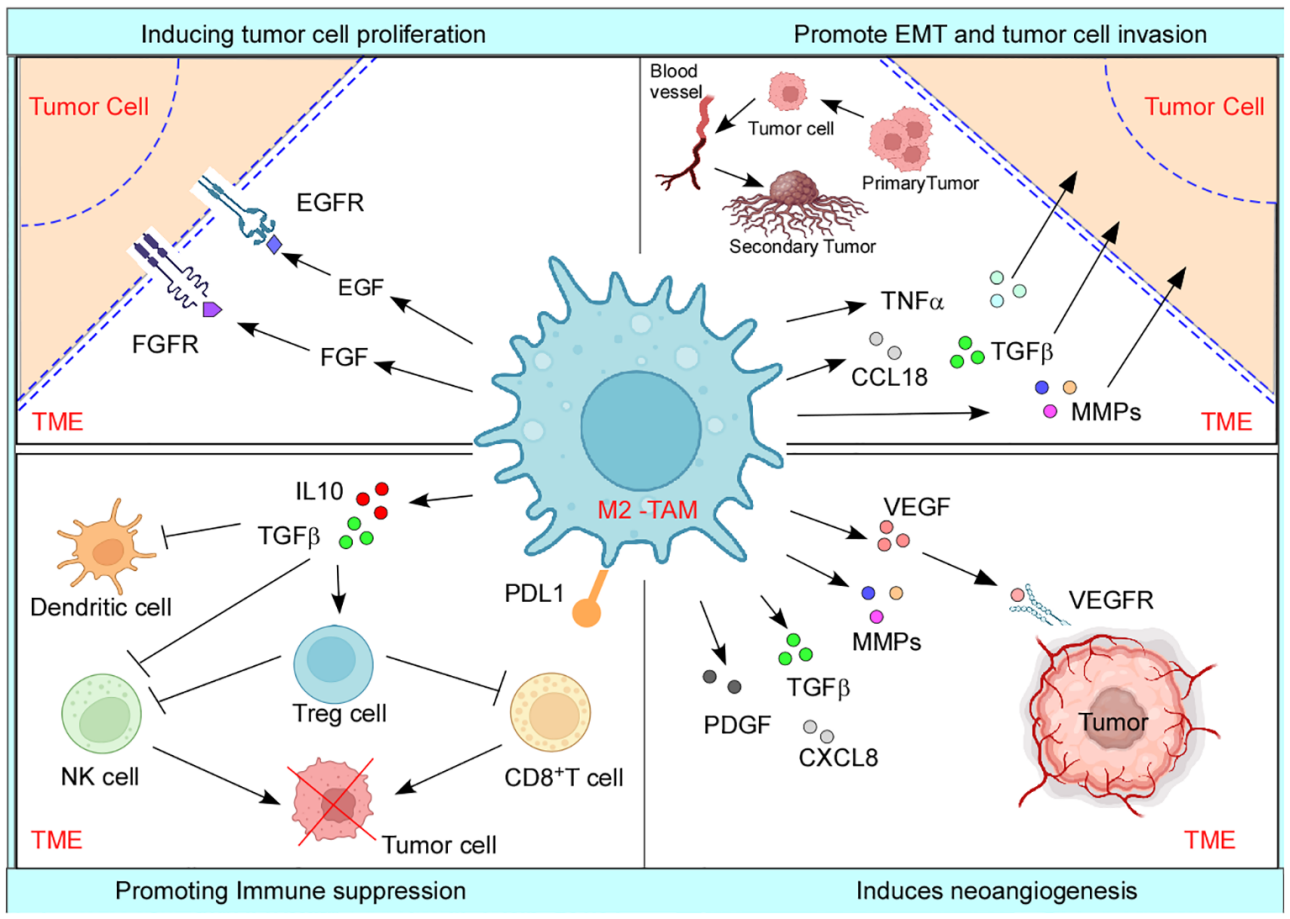
M2-TAM promoting different aspects of tumor development: TAMs encourage increased tumor cell proliferation by secreting tumor cell proliferating growth factors such as EGF and FGF. TAMs cause neoangiogenesis and lymphangiogenesis by releasing various pro-angiogenic factors such as VEGF, PDGF, TGFβ, MMPs, and CXCL8. TAMs also lead to increased EMT and extracellular matrix remodeling by releasing factors such as CCL18, TGFβ, MMPs, and TNFα, which ultimately causes metastasis and secondary tumor formation. TAMs negatively affect the functions of NK cells, DCs, and cytotoxic T cells and promote immunosuppression by actively playing a role in the recruitment of Treg cells in the TME. Created with BioRender.com.

Several growth factor receptors, such as EGFR and FGFR proteins, along with their associated signaling pathways, are frequently upregulated in a multitude of human malignancies, which ultimately result in heightened cellular proliferation and enhanced survival rates (106). TAMs employ this mechanism by producing growth factors like epidermal growth factor (EGF) and fibroblast growth factor (FGF) to facilitate the activation of downstream signaling pathways, subsequently leading to enhanced survival, migration, metastasis, and suppression of apoptosis in cancer cells (106) (**Figure 2**). TGFβ is widely recognized for its dichotomous function in the advancement of cancer, while serving as a suppressor of tumor growth during the initial phases and subsequently as a promoter of tumor development in later stages. Loss of ability in late-stage cancer to respond to cytostatic functions of TGFβ again results in increased cell proliferation, survival, angiogenesis, and immunosuppression (107). Stemming evidence indicates that TAM‐derived TGFβ promotes colorectal cancer progression through HIF1‐TRIB3 signaling (108). Additionally, TAMs secrete large amounts of immunosuppressive cytokine IL10, which prevents tumor cell-killing activity of CD8^+^ T cells, Th1 cells, and NK cells (109), thereby limiting cytotoxicity of the TME to help in tumor growth. Also, TAM-derived adrenomedullin interacts with endothelial cells to promote tumor growth through activation of the eNOS signaling pathway in a paracrine manner (110). Therefore, the development of tumors is critically related to the molecular cues shed by TAM.

### 5.2 TAMs cause neoangiogenesis and lymphangiogenesis

The process of metastasis requires vascularization in primary tumors, which otherwise grow only up to 2–3 mm^3^ in size (106). TAMs encourage the sprouting of new blood vessels to provide oxygen and nutrients to proliferating cancer cells, described in animal models of ovarian cancer, cervical cancer, prostate cancer, breast cancer, and melanoma (111). Although tumor cells themselves provide the required stimuli to begin vascularization, the process is hindered in the absence of TAMs. Zeisberger et al. found that depletion of TAMs with clodronate encapsulated in liposomes (clodrolip) reduced blood vessel density in the tumor tissue (112). These results validate the idea that TAMs present in TME promote neoangiogenesis in tumors. Perpetuation of hypoxia and the formation of dysfunctional vessels are the by-products of tumor angiogenesis, as well (113).

TAMs are known to release a diverse array of molecules that are essential for the process of neoangiogenesis, which, in turn, plays a critical role in the onset of metastasis. For example, TAMs release growth factors such as VEGF, PDGF, TGFβ, and FGF, that promote angiogenesis (**Figure 2**) in numerous cancers, namely, gliomas, squamous cell carcinomas of the esophagus, breast, bladder, and prostate carcinomas (114, 115). Interestingly, angiogenic factors govern the process in a macrophage subtype context too; for instance, FGF signaling controls M2a-induced angiogenesis while placental growth factor (PlGF) signaling controls M2c-induced angiogenesis (55). Moreover, TAM-derived MMP1, MMP2, MMP3, MMP9, MMP12, plasmin, and urokinase plasminogen contribute to the cause of angiogenesis by degrading the ECM (116), which might later aid in metastasis. Accumulation of TAMs in tumor hypoxic regions and their adaptability to the low-oxygen microenvironment is particularly fascinating as they over-express pro-angiogenic factors (VEGF, pFGF, and CXCL8) and glycolytic enzymes, regulated by hypoxia-induced transcription factors HIF1 and HIF2 (109). In addition to this, HIF1-dependent chemokine CXCL12 is released by TAMs and serves as a potent chemoattractant to CXCR4-expressing endothelial cells, causing migration of endothelial cells, further helping neoangiogenesis (117). Furthermore, by closely collaborating with endothelial cells at the angiogenic branching points, M2-like macrophages aid the development of blood vessels by encouraging endothelial cells to condense into tubes, forming a tubular network (55). Cumulatively, TAMs and hypoxia both regulate one another in a positive feedback loop as hypoxia drives TAM polarization (97) and, on the other hand, TAMs favor hypoxia. TAM-led neoangiogenesis often amounts to abnormal blood vessel formation with irregular blood flow, laying the ground for hypoxia (118). In addition, such leaky blood vessels give way to tumor cell intravasation and metastasis (97). Interestingly, it has been recently reported that M2-derived exosomes transferring miR-193a-5p lead to tumor progression by endorsing vascular mimicry (119).

Similar to angiogenesis, lymphangiogenesis provides an additional avenue for malignant cells to travel through the body and establish footholds in other areas, hence predicting a poor clinical prognosis. TAMs promote lymphangiogenesis through the VEGFC/VEGFD-VEGFR3 signaling pathway (120) and frequently produce MMP9, leading to the development of lymphatic vessels. CD11b^+^ macrophages express lymphatic EGFs and induce lymphangiogenesis, either by trans-differentiating and directly incorporating into the endothelial layer or by stimulating the division of pre-existing local lymphatic endothelial cells (121). As a consequence, TAM-mediated vascular growth ensures survival of tumor cells at the local site along with paving the path for distant metastasis.

### 5.3 TAMs lead to epithelial–mesenchymal transition and extracellular matrix remodeling

Epithelial–mesenchymal transition (EMT) is yet another cardinal aspect abetting metastasis wherein epithelial-like cancer cells lose the capacity for cell–cell adhesion and adopt a fibroblast-like phenotype with invasive and migratory properties, enabling cancer cells to leave the primary tissue site and enter the bloodstream or lymphatic vessels to infiltrate other organs (122) (**Figure 2**). Over two decades ago, Gorelik et al. conducted an *in vivo* study where they showed that TAMs play a significant role in tumor metastasis (123). Intravenous administration of murine tumor cells resulted in a notable augmentation in the TAM population at the site of lung tumor nodule development. Subsequent investigation has elucidated that EGF secreted by TAMs engages with CSF1 released by tumor cells facilitating the migratory capabilities of the tumor cells (124).

Hagemann et al. established that co-culturing TAMs with tumor cells promotes the expression of MMP2, MMP7, and MMP9 in a TNF*α*-dependent manner, causing the breakdown of ECM proteins and thereby helping metastasis (125). Furthermore, Seth et al. demonstrated that MMP7 could also encourage tumor metastasis through NF*κ*B-RANKL signaling (126). MMP9 from M2-like TAMs causes collagen proteolytic clearance and lysosomal degradation. TAMs are also involved in the overexpression and reorganization of several collagen fibers, including collagen I and collagen VI, resulting in ECM deposition near aggressive tumor cells (127). Thus, TAMs remodel ECM surrounding cancer cells, which may protect them from external assaults while also degrading the ECM matrix to facilitate metastasis. TNFα and TGFβ produced by TAMs aid SNAIL and ZEB1 expression by activating the NFκB and β-catenin pathways respectively, adding to the EMT cause (128). Also, a positive feedback loop generated by tumor-shed GMCSF activates TAM to release CCL18, supporting EMT (129) (**Figure 2**). Fascinatingly, it has also been demonstrated that decreased expression of E-cadherin, a tumor-suppressor protein that acts antithetical to EMT and metastasis, is associated with increased expression of the M2 marker CD68 (130).

In breast carcinoma and lung adenocarcinoma models, TAMs are implicated in active ECM remodeling by working in close conjunction with CAFs to increase tumor cell intravasation (131). CAFs and TAMs have a mutually beneficial relationship that orchestrates tumor growth and progression (132). Prostate CAFs, for example, have been observed to be involved in monocyte recruitment and M2 phenotypic skewing. M2-like TAMs, on the other hand, trigger the mesenchymal transition of CAFs, hence increasing their pro-tumor activity. Cancer cells collectively influence the TAM–CAF interaction, resulting in increased metastasis and angiogenesis (133). When compared to normal breast-derived fibroblasts, invasive breast cancer-derived CAFs were able to drive monocytes towards the immunosuppressive M2 type. CAFs induced migration-related proteins in cancer cells while also influencing macrophage phenotype (134). The co-existence of CAFs with high numbers of TAMs was positively correlated in breast and oral cancers, serving as a marker for poor patient survival (132, 134). Similarly, CAF shed GMCSF and IL6, which both promote the pro-tumor TAM and cancer invasiveness (132). TAM-shed osteopontin also stimulates CAF to shed osteopontin to promote ECM modification and migration in HCC (135).

Regular tissue fibroblasts, secrete a diverse range of ECM ingredients such as collagen, elastin, fibronectin, and proteoglycans to build a network of fibers that acts as a barrier protecting tumor cells from mechanical, pharmacological, and immunological stress (132). CAFs, on the other hand, secrete fibroblast activation protein, a serine protease that remodels the ECM by rebuilding collagen and fibronectin into parallel orientation for improved pancreatic cancer cell motility and invasiveness (136). CAFs are also known to operate as leading cells that modify the ECM matrix and are closely followed by tumor cells that travel the paved way for them, causing metastasis (137). CAF expression of MMP11, MMP2, MMP9, and MMP21 in various malignancies is associated with a significant risk of tumor relapse (132). Furthermore, CAF-mediated ECM breakdown aids TAM infiltration into the tumor site. CAF-released ECM components likewise show an M2 bias (138, 139). TAM trafficking in TME is linked to the release of hyaluronan by stromal cells. Hyaluronan synthase-2 (HAS2) depletion in fibroblasts resulted in reduced macrophage recruitment, increased angiogenesis, and lymphangiogenesis at the tumor site (140). Thus, CAFs play an important role in regulating TAM polarization and mobilization, as well as encouraging ECM reorganization, which contributes to cancer migration.

Therefore, promising pieces of evidence describe TAM hallmarks and explore emerging mechanisms that contribute to their pathophysiological adaptations that promote tumorigenesis by fostering angiogenesis, invasion, and metastasis (141).

## 6 TAMs interact with other immune cells and cause immunosuppression in the TME

Immunosuppression in tumors makes it easier for cancer cells to evade immune surveillance (142). Studies have linked the role of different cytokines and molecules secreted by TAMs, such as TGFβ, IL10, and arginase-1, along with the activation of certain signaling pathways to play a significant role in immunosuppression (7). TGFβ influences macrophage polarization in TME during the innate immune response, favoring the M2 phenotype, which again encourages the synthesis of additional TGFβ (143). TGFβ also prevents the cytolytic activity of NK cells (7), while both TAM-derived IL10 and TGFβ inhibit the maturation and functioning of dendritic cells (DCs), along with inducing apoptosis. This results in reduced antigen presentation and dampened adaptive immune response (144). TGFβ regulates the production of various cytolytic genes as well, including granzyme A, granzyme B, IFNγ, and FAS ligand, thereby impairing the anti-tumor activity of CD8^+^ T cells during the adaptive immune response and ultimately resulting in pro-tumorigenic TME (7). Moreover, TAMs can directly promote cytotoxic T-cell exhaustion. Infiltrating CD8^+^ T cells are observed to have greater effector potential after *in vivo* TAM reduction (145). TGFβ is also associated with supporting the differentiation of Treg cells, a powerful immunosuppressing arm of the TME (146). TAM-mediated Treg recruitment has been reported in ovarian cancer, nasopharyngeal carcinoma, and liver cancer. Such recruited Tregs deactivate cytotoxic T cells against cancer (145). On the other hand, TAM-derived IL10 suppresses the expression of anti-tumor cytokines IL12 and IFNγ, involved in naïve T-cell differentiation, and further inhibits the function of cytotoxic T cell, NK, and lymphokine-activated killer cells (**Figure 2**) (145). It has been shown that arginase-1, a marker for M2 macrophages, converts L-arginine into polyamine and proline, causing the TCR signal to be dysregulated, and resultantly causing CD8^+^ T-cell anergy (147). Additionally, cues provided by TAMs lead to the expansion of GR1^+^CD11b^+^MDSCs, which overexpresses iNOS, arginase, and TGFβ, directly contributing to immunosuppression (148). Multiple TAM-produced chemokines have been related to immunosuppression as well. For example, TAM-derived CCL17/CCL22 significantly aids in infiltration of Tregs to the TME through the chemokine receptor CCR4 (149). CCL18 produced by TAM attracts naïve T cells to the tumor site, resulting in T-cell anergy (114).

Another effective mechanism M2-TAMs employ to deplete the anti-tumoral immune response and carry forward its immunosuppressive streak is by displaying an amplified expression of immune checkpoint molecules PDL1 and cytotoxic T-lymphocyte antigen 4 (CTLA4) ligand (150). Heightened levels of these molecules are correlated with poor prognosis in various cancers such as HCC and glioblastoma (151); therefore, they present themselves as secondary targets for current immunotherapies, by using ICI such as pembrolizumab or nivolumab (152). This discussion clarifies how M2-TAM promotes different aspects of tumor development (**Figure 2**). Interestingly, inflammation in the TME is also associated with cancer progression. TAMs and monocytes contribute to this inflammatory process by the generation of IL21^+^ T follicular helper cells (TFH cells). This creates a local environment suitable for the differentiation of plasma cells and M2b macrophages, which promotes cancer progression (153). TAMs also establish an inflammatory milieu via releasing numerous pro-inflammatory cytokines such as CXCL8 in endometrial cancer, IL6 in breast cancer, and IL1β in pancreatic cancer, which ultimately have correlated with poor prognosis (145).

However, focusing on TAMs and their interaction with only cancer cells and immune components is addressing half of the story. The other half is the interplay of CSC–TAM, which may be the cardinal cause of tumor progression and survival, owing to the tumor-initiating, metastatic, and relapse properties of CSCs and the TAMs that contribute to the major population of TME and their pro-cancerous inclination.

## 7 Cancer stem cells and TAMs

As previously pointed out, the rare subpopulations of CSCs are the drivers of an entire tumor mass due to their tumor-initiating properties. According to the CSC theory, CSC resides on top of the hierarchal model in tumor development wherein, much like somatic stem cells, they undergo asymmetrical division to generate differentiated NSCCs sculpting the tumor, as well as self-renew to maintain itself (154, 155). CSCs not only develop a tumor at its primary site, but they also metastasize to the secondary site to create a new tumor (4, 156). All disseminated cells are not able to make it through the metastatic journey or can successfully break away from ECM from the primary site (157, 158). The exceptional properties of CSCs to modulate ECM and undergo EMT, and their inherent quiescent nature, make them sturdy for this tedious journey (157, 159). A previous report from our group has elucidated how intrinsic non-migratory CSCs give rise to metastatic CXCR4^+^ CSCs, which indeed are responsible for the seeding of new tumors at secondary sites (160). Additionally, resistance to chemo- or radiotherapy and causing further relapse to the same or distant sites are attributed to CSCs as well (161, 162). CSCs, but not NSCCs, successfully escape these treatment regimens owing to their self-protecting non-proliferating quiescent state and overexpression of drug resistance pumps (163, 164). Intriguingly, chemotherapy leads to an increase in this CSC subpopulation which may later “recreate” more aggressive tumors after therapy removal, explaining the failure of eradicating cancer in many cases (165). However, the pivotal role that CSC plays especially in tumor initiation, metastasis, and tumor recurrence would not have been possible, if it did not evade the anti-tumor immune response first and further carry out immune-editing in its favor (166).

CSCs hamper the anti-immune components of their niche to ensure their existence. For example, CSCs inhibit anti-tumor NK cells by inducing apoptosis on one hand, while moderating their cell surface receptors to avoid detection on the other (23, 167). Similarly, CSCs avoid CD8^+^ cytotoxic T-cell immune surveillance by downregulating MHC-I expression and in turn, impede their growth by selectively augmenting the inhibitory checkpoint receptors (23, 168). Additionally, CSCs promote a pro-tumor environment by recruiting immunosuppressive MDSCs and T-regulatory cells, known to release cytokines and bring about metabolic changes that not only help in CSC maintenance but also empower their selective survival over the others (14, 169, 170). The existence and the position of TAMs are observed to be positively correlated with that of CSCs. Numerous reports suggested that the distribution of infiltrating TAMs in numbers and being in the vicinity of CSCs are also directly associated with histological grade and the number of CSCs present (7, 171). Similarly, TAM accumulation was also clearly evident within the hypoxic regions of tumors (13), which correlates with the existence of CSCs as well in these hypoxic regions (172). Further studies suggested that CSCs and TAMs, when introduced simultaneously in wild-type syngeneic mice models, depict more efficient tumor initiation and metastatic activities (173). Supporting the positive reciprocal cross-regulation of CSCs and TAMs, another finding showed heightened tumor formation capability in xenograft models when TAMs were co-cultured with hepatocellular CSCs first (2, 174). Considering that TAMs constitute the dominant proportion of the tumor milieu and their close partnership with CSCs, their dialog in TME needs to be understood further.

### 7.1 CSC effect on TAMs

**Figure 3:**
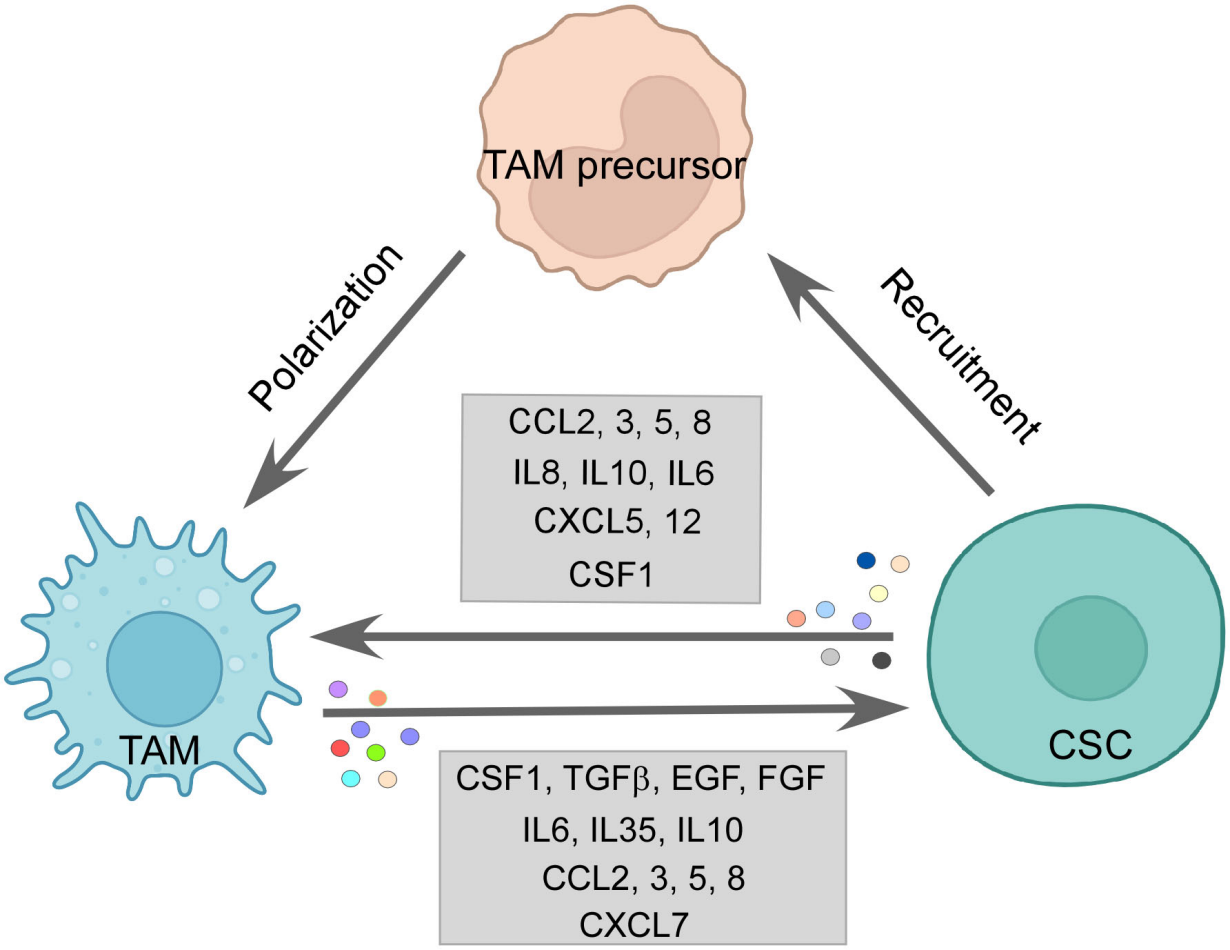
The CSC–TAM crosstalk: CSCs employ various modalities to recruit M2-TAMs, such as CCL2, CCL5, CCL8, and CSF1. M2-TAMs are also polarized in the tumor site by CCL2, CCL5, IL6, IL10, and CSF1 by CSCs. Reciprocally, TAMs promote CSC function by releasing CSF1, TGFβ, IL6, IL10, IL35, EGF, and FGF among others, thereby, maintaining a vicious loop for sustenance of one another. Created with BioRender.com.

CSCs release chemotactic factors such as CCL2, CCL3, CCL5, CCL8, CXCL5, and CXCL12 dynamically to recruit macrophages or their precursors (7, 175). It has been observed in a multitude of cancers that CCL2 is expressed in higher amounts by CSCs than non-CSC counterparts. Along with this, higher expression of CCL2 correlated with higher engagement of TAMs, especially the M2 subtype to the tumor site, while blocking of the same impaired the M2 population recruitment (175, 176). Interestingly, the production of CCL2 is influenced by the protein TWIST, which is known to be upregulated in CSCs (177). CCL5 is seen to be exclusively secreted by CSCs (175). Although photodynamic therapy (PDT) elicited CCL8, which actively brought about an M1 anti-tumor response in cutaneous squamous cell carcinoma (178), CCL8 is also reported to enhance stemness in cancers (179). Both CXCL5 and CXCL12 are expressed by CSCs, which readily encourages TAM infiltration. CXCL5 being downstream of Sox9 attracts both neutrophils and macrophages, while CXCL12 activates CXCR7, which regulates metastasis in breast cancer as well as M2 infiltration (175). Interleukins such as IL33 are also secreted by CSCs to create a pro-tumorigenic environment by attracting TAMs to their site (180). Some soluble factors, such as CSF1 and VEGFA, are also reported to be involved in TAM recruitment. Glioma CSCs are reported to release CSF1 and cytokines not only to recruit macrophages but also to polarize them to an immunosuppressive type (7, 175) (**Figure 3**).

Similarly, CCL2 and CCL5 released by CSCs contribute to both macrophage infiltration and skew them towards the tumor-supporting M2 subtype (181, 182) (**Figure 3**). A multitude of cytokines participate in this process of altering the functional state of the macrophages in favor of CSCs; for example, IL6, IL8, and IL10 released by the CSCs are all reported to employ STAT3 signaling to convert TAMs to M2 phenotype (175). IL10 being highly expressed in CSCs is also known to engage NFκB to polarize M2-like macrophage (183). Moreover, IL4 and IL13 released by the CSCs also bring about the pro-tumor state of macrophages (12). Again, CSF, a recruiter for macrophages, also supports the M2 phenotype along with the CSC-released immunosuppressive cytokine TGFβ (184). CSC-derived exosomes are reported to carry out the same effect as well. For instance, glioblastoma stem cell-derived exosomes skewed monocytes to pro-tumor M2 macrophage by transferring them with STAT3, the activation of which we have already seen to induce the pro-tumor TAMs (185). Moreover, CSCs can push macrophages toward M2 by direct contact-dependent mechanisms as well (175), therefore elucidating the varied ways CSCs use to exercise their immunomodulatory arm.

Apart from manipulating immune cells on its side, CSCs also evade immune-mediated destruction. CSCs express low levels of MHC class molecules and co-stimulatory receptors, which may otherwise trigger immune responses (186). Additionally, immune checkpoint ligands PDL1 and B7-H3 are overexpressed on CSCs to limit the growth of immune cells (12). Another notable immune evasion mechanism employed by CSC, especially against phagocytosis, is the overexpression of “don’t eat me marker” CD47, the interaction of which with SIRPα on M1 macrophages stills the phagocytosis process (12, 13). Furthermore, it has been observed that M2-like TAM expresses SIRPα at a noticeably higher level and interacts with the upregulated CD47 in CSCs (187, 188).

### 7.2 TAM’s effect on CSCs

Reciprocally, the M2-TAMs help in CSC maintenance, growth, and EMT as well. Incidentally, the chemokines used by the CSCs to promote TAMs are also secreted by TAMs to help CSCs (12). CCL2 via the β-catenin pathway upregulated CSC-related stemness genes along with increasing ALDH and CD44 expressing CSC pool in breast cancer (181). Also, CCL3 released by TAMs induced migration and invasion characteristics (189). Similarly, CCL5 and CCL8 secretion by TAMs promoted stemness and invasion features in glioblastoma (179, 190). CXCL7 releasing TAMs, recruited by glioma, helps in regulating the stemness in such cancer cells (191). In HCC and breast cancer, IL6 from TAMs activates the STAT3 pathway to promote CSC population, migration, and angiogenesis (174, 192). TAMs also shed IL10 and IL35, which endorses the CSC nature. For example, IL10 from M2-TAMs induces the upregulation of stemness genes in lung cancer. Inhibition of the same presented as a potential therapeutic target (175). Therefore, observably IL6 and IL10 act in a pro-tumor immunosuppressive loop in TME where both M2 and CSCs secrete them, supporting the growth of one another. Interestingly, not only M2 but M1-TAMs are also reported to secrete IL6 and IL12, which nudge the tumor cells towards CSC phenotype (175). Moreover, factors like TGFβ, EGF, and FGF secreted by TAMs also skew cancer cells to CSCs. Fascinatingly, CSF1 and EGF establish a feedback paracrine loop between CSCs and TAMs, where CSF1 recruited TAMs shed a higher amount of EGF leading to stimulation of STAT3/SOX2 pathway in breast cancer cells (12, 193) (**Figure 3**). Talking of feedback mechanisms, CSC encourages TAM to release MGF-E8, which, via STAT3 and sonic hedgehog pathways, induces CSC-led tumor formation and resistance to chemotherapy drugs (194). M2-derived extracellular vesicles deliver miR-21-5p, which maintains CSC stemness and characteristics in pancreatic cancer (195).

TAMs impact CSC growth by contact-dependent mechanisms as well. Metastatic CSCs express HAS2, which facilitates the interaction between CSCs and TAMs, leading to secretion of PDGF-BB by TAMs. This factor, in turn, orchestrates the secretion of FGF7 and FGF9 from bone stromal cells, enabling CSC growth (196). The physical interaction of CD90 and Ephrin type A expressed on CSCs with their respective ligands on macrophages activates signaling pathways, thereby enhancing high tumorigenicity and metastasis by CSCs (173). Another juxtacrine interaction of LSECtin on TAMs and BTN3A3 receptor on CSCs drives tumor stemness (197). GPNMB on TAMs binds to the CD44 of tumor stem cells, eliciting a response of IL33, which is a proponent of CSC growth. In addition, IL33 instigates macrophages to release pro-CSC cytokine TGFβ (12). Consequently, the dynamic feed-forward equation between CSCs and TAMs drives the population of each other and creates an immunosuppressive environment, which consequently supports the survival of both populations (**Figure 3**).

## 8 Therapeutic intervention

Considering the crucial role played by TAMs in promoting pro-cancerous TME, targeting these TAMs becomes imperative for alleviating their tumor-reinforcing arm for better anti-cancer therapy outcomes. This can be achieved by depletion of TAM, blocking TAM infiltration, and polarizing TAMs to M1 phenotype, which resultantly leads to activation of anti-tumor activity, overcoming TAM-mediated immunosuppressive TME and engaging their phagocytosis potential (2, 11).

### 8.1 Depletion and inhibiting infiltration of TAMs in TME

**Figure 4:**
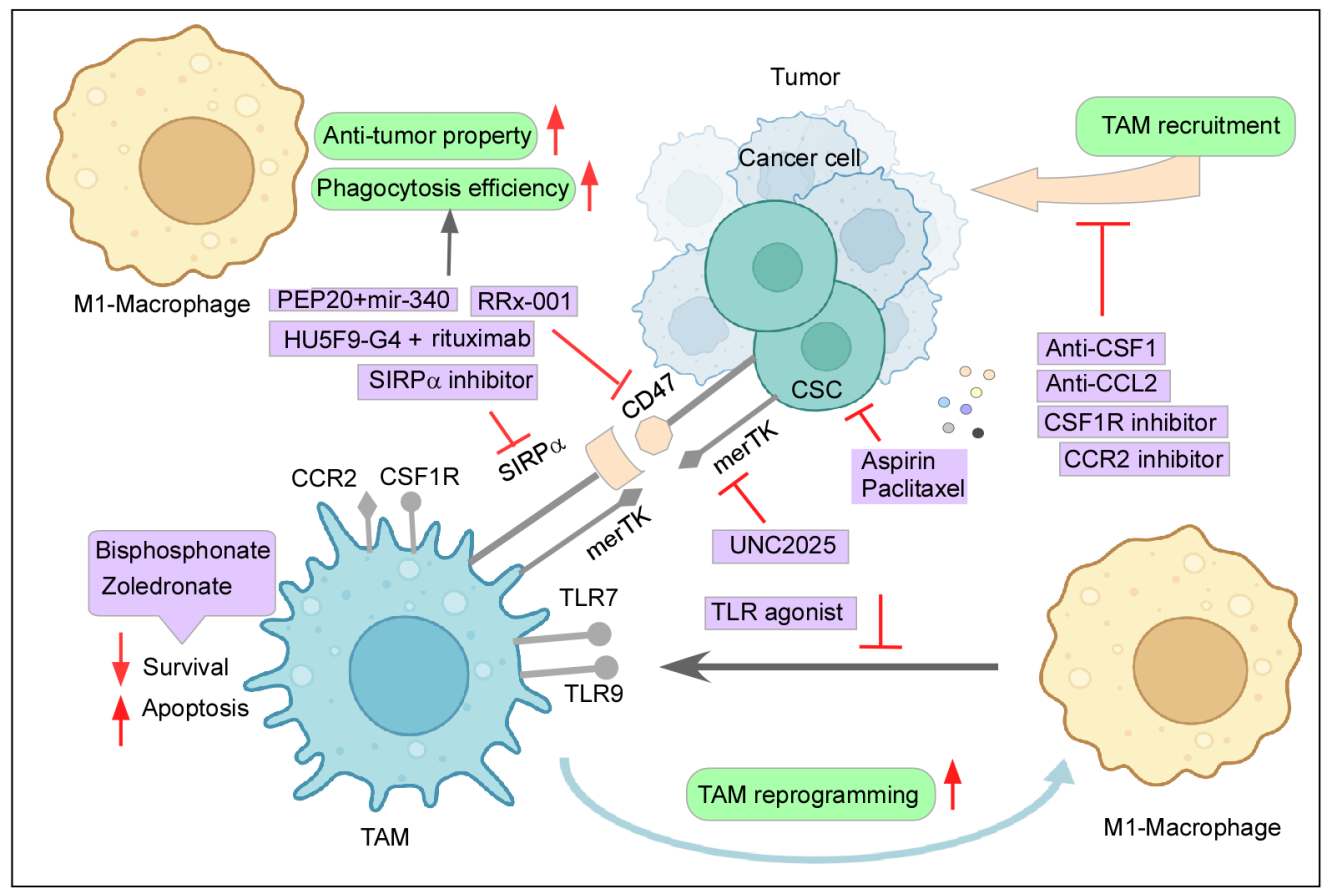
Therapeutic intervention to curb the tumor–TAM interplay: Mechanisms to inhibit the cancer-TAM crosstalk include depletion and blocking M2-TAM recruitment, re-programming of M2-TAMs, engaging phagocytic activity, and dysregulating CSC–TAM crosstalk. Created with BioRender.com.

Bisphosphonates are known to induce TAM apoptosis and suppress the migration and infiltration of TAMs (11). Zoledronate, a class of bisphosphonate, can be specifically delivered to attack TAMs by nanoparticle systems and has shown promising results of reduced immunosuppressive effect and tumor growth in mice models (11, 198). Chronic stress-induced macrophage infiltration and higher expression of PDGF-AA in orthotopic ovarian cancer models create an inflammatory environment with negative implications. This stress-induced unfavorable effect can be reversed by Zoledronic acid, a macrophage-depleting drug (199). Furthermore, macrophages generated from the peritoneal cavity of mice with subcutaneous pancreatic cancer promoted tumor growth and metastasis, which Zoledronic acid successfully inhibited (200). Zoledronic acid coupled with doxorubicin was effective against breast and peritoneal TAMs in both *in vitro* and in animal models (201) (**Figure 4**). As seen earlier, CSF plays a paramount role in TAM recruitment and survival, and therefore, blockading of the CSF1/CSF1R axis can reduce monocyte recruitment to the tumor site and its differentiation while further hampering the survivability of the existing TAMs (6, 11). Interestingly, monoclonal antibodies and small-molecule inhibitors targeting CSF1R have been reported to result in a decreased number of TAMs and an increased ratio of CD8^+^/CD4^+^ T cells in the tumor (9, 202). TD-92, an erlotinib derivative targeting CSF1R, not only depletes TAM at the tumor site but also increases the efficacy of anti-PD1 therapy (203). Monoclonal antibodies against CSF1, Lacnotuzumab (NCT02435680), with chemotherapy showed improved patient responses compared to chemotherapy alone in advanced triple-negative breast cancer in a recent randomized phase II clinical trial (204). Similarly, LY3022855 (NCT01346358) showed decreased TAM levels in solid refractory tumors (205) (**Figure 4**). Despite the few upsides reported by this therapy, numerous accounts state otherwise. Long-term blockade of CSF1R led to PI3K activation, which led to therapy failure (206). Impairing CSF1R axis may also have a limited durable response due to infiltration of Tregs in the tumor milieu and inhibition of TRMs, which are crucial elements for a body’s immune equilibrium. Moreover, in contrast to some previous studies, attenuating CSF1R along with PD1 immunotherapy failed to produce significant positive outcomes in patient clinical trials (6, 207).

Similarly, the CCR2–CCL2 axis also provides itself as an attractive therapeutic strategy due to its reported role in active monocyte recruitment. Aiming this signaling pathway has been shown to reduce tumor growth, decrease the incidence of M2-TAM at the primary site and metastatic sites as well, and elicit an anti-tumor T-cell response (11, 208). In glioblastoma animal models, CCR2–CCL2 inhibitors effectively reduced the M2-TAM infiltration at the tumor site, improved survival, and demonstrated a better outcome of combinatorial anti-cancer therapies (11, 209). A clinical trial (NCT01413022) involving CCR2 inhibitor PF-04136309 in association with chemotherapy was observed to be safe and tolerable for locally advanced pancreatic cancer (210). When compared to chemotherapy alone, a CCR2 antagonist, CCX827 (NCT02345408), coupled with a chemotherapeutic drugs, FOLFIRINOX, improved patients’ overall survival in advanced pancreatic cancer (211). Similarly, Carlumab (NCT01204996), a monoclonal antibody against CCL2, displayed transitory effectiveness against tumor (212) (**Figure 4**). However, the withdrawal of the anti-CCL2 treatment regime exacerbated monocyte infiltration and metastasis (213). In addition to this, blocking of this axis did not affect the already recruited TAMs as they continued to aid tumor progression (18). Abating other monocyte recruitment strategies has delivered some positive results. Restricting the CCL5/CCR5 axis-mounted anti-tumor response lowers TAM trafficking and reduces tumor growth (214). Similarly, attacking IL8/CXCR2 generated higher efficacy towards PD1 treatment (215). Targeting the CXCL12–CXCR4 pathway along with chemotherapy also provided some relief in tumor burden by reducing M2-TAMs and tumor revascularization (216). Recent clinical trials employing CXCR4 antagonist LY2510924 in combination with durvalumab have shown a safe and manageable response in a phase 1 clinical trial (NCT02737072) with advanced refractory solid tumor (217). Furthermore, the CXCR4 inhibitor motixafortide in combination with pembrolizumab (anti-PD1 antibody) and chemotherapy demonstrated excellent benefits in patients with advanced pancreatic cancer (COMBAT/KEYNOTE-202 study NCT02826486) (218).

### 8.2 Reprogramming TAMs to anti-tumor M1 phenotype

Reprogramming M2-TAMs to pro-inflammatory anti-tumor M1 subtype also presented itself as an attractive targeting strategy in this regard. Inhibiting CSF1R not only blocks TAM recruitment but also skews M2 to M1 phenotype, which effectively resulted in retarded tumor growth, inducing cytotoxic T-cell response and better prognosis with combinatorial chemotherapy (219). TLR agonists also push the TAM environment towards M1, thereby creating inflammatory cytokine response, resulting in a reasonable effect on solid tumors. The TLR7 agonist imiquimod is FDA-approved and is effective against basal cell carcinomas (220). Imiquimod (NCT00899574) showed partial response in patients with breast cancer involving skin metastases (221). A TLR3 stimulant packaged in a nanoparticle has been shown to reduce tumor growth by excessive production of TNFα and NO (9, 222). Another instance is the TLR9 agonist, lefitolimod, which favors M1-TAM and generates other anti-tumor responses (223, 224) and has been the topic of study in multiple clinical trials such as NCT02668770 (225). Another method of activating an M1 response is by using CD40 agonists, which are proven effective against prostate cancer progression, as well as sensitizing otherwise resistant tumors towards chemotherapy (226). An ongoing clinical trial (NCT03214250) using a CD40 agonist, APX005M, along with chemotherapy has shown clinical efficacy against advanced pancreatic cancer (227). Other mechanisms such as PDT and upregulation of miR-130 and miR-33 cause M1 polarization and effective tumor regression (11, 228).

Another major advantage of reprogramming TAMs to M1 for anti-cancer therapy is to exploit the phagocytic potential of M1 and consequently attack the CD47–SIRPα axis, already discussed in its involvement in immune evasion (2, 11). RRx-001, a multi-functional drug used in clinical trials such as NCT02518958, not only can promote M1-TAM phenotype but also can be used as an inhibitor of CD47 on cancer cells and SIRPα on macrophages (229, 230). The use of anti-CD47 mAb treatments also revealed favoring of the M1-TAM population with increased phagocytic activity. Multiple strategies have been devised to either block CD47 or SIRPα, and have proven effective in anti-cancer therapy. For example, a clinical trial (NCT02953509) CD47 antagonist HU5F9-G4 along with rituximab demonstrated a 50% response rate (inclusive of complete and partial) in non-Hodgkin’s lymphoma (231, 232) (**Figure 4**). Even when cancer cells were resistant to anti-HER2 trastuzumab, anti-CD47 (magrolimab) combined with trastuzumab dramatically reduced HER2^+^ breast cancer with heightened efficacy (233). Likewise, a polypeptide PEP-20 and microRNA miR-340 have been reported to target CD47 and enhance macrophage-mediated phagocytosis (234, 235). Similarly, AB-21, a blocker of SIRPα, has generated identical results (236).

Phenotype switching of TAMs along with exerting anti-tumor effect also averts the immunosuppressive role of M2. A deadly reciprocal loop by TGFβ in immunosuppressive TME, induced and maintained by macrophages and cancer cells, targeted along with PD1 resulted in successful activation of anti-tumor machinery (237). A clinical trial (NCT03459222) employing ICI and targeting IDO1 (238) is now underway in advanced solid tumors; IDO1 contributes to an immunosuppressive environment and is released by TAMs (50). Moreover, curbing scavenger receptor MARCO expressing TAMs has also helped in reversing immunosuppressive TME (239).

However, polarization to M1-TAMs produced an anti-inflammatory response but failed to reduce tumor size in patients according to a couple of studies. This may be due to the re-skewing of M1-TAMs to M2 because of the environmental cues present in TME or due to M1-mediated tumor progression (6). In HCC, M1 macrophage encourages EMT by secretion of IL35 (240). These instances might explain why reprogramming of TAMs provided limited promising follow-ups. Therefore, to eliminate these roadblocks in TAM-related therapy, genetically engineered macrophages like CAR-T were developed. Engineered macrophages transduced with stable IL21 and decoy TGFβ receptors present themselves as potential cancer immunotherapy (241). Furthermore, anti-CD19 and anti-CD22 CARs in murine macrophage models demonstrated activation and enhanced phagocytic activities. Chimeric Antigen Receptor-expressing Macrophages (CAR-M) led to a significant reduction in cancer progression and improved overall survival in mice models (6, 242). CAR-M administration led to a stable pro-inflammatory subset induction; while M2 was not able to change the CAR-M to an immunosuppressive type, CAR-M induced these M2 to M1 phenotypes. CAR-M was able to notably induce other anti-tumor immune responses as well, bolstering the future role of CAR-M in immunotherapy (6). A clinical trial (NCT04660929) is underway employing CAR-M Anti-HER2 receptors against HER2-overexpressing tumors, with the intention of producing beneficial results (243). This discussion signifies the importance of therapeutic intervention to curb the tumor–TAM interplay for successful tumor regression (**Figure 4**).

It would be not out of context to mention that therapies attacking TAM–cancer cell crosstalk may provide initial tumor regression; however, for actual elimination of the “root cause” of cancer, CSC–TAM interaction becomes crucial to target, as CSCs are attributed for their pivotal roles in drug resistance, relapse, and metastasis.

### 8.3 Therapeutic intervention towards CSC–TAM crosstalk

Since CSC status is directly correlated with TAMs, it is observed that the depletion of TAMs by inhibiting the CCL2/CCR2 pathway led to a decreased incidence of CSCs in pancreatic ductal adenocarcinoma (244). Moreover, aspirin has been effective in reducing the recruitment of M2 TAMs by targeting CCL2, as well as CSC marker NANOG along with declining their sphere formation ability and the expression of CSC marker genes CD90 and NANOG in esophageal cancer (245). Highly metastatic CXCR4^+^ CSCs were targeted by CXCR4 antagonists like Plerixafor, inhibiting the CXCL12/CXCR4 axis and curbing macrophage infiltration with CSC metastasis (246). Carrying this line of thought, aiming CSF-1R for the depletion of TAMs along with combinatorial chemotherapy has proven effective against cancer cells, and might also affect the CSC population. Consequently, such therapeutic measures act bidirectionally by simultaneously taking action on both ends of CSC–TAM crosstalk (175). CSCs and TAMs both act as a source and sink for IL6, wherein IL6 promotes STAT3 and helps maintain the immunosuppressive nature in the case of TAMs and also enhances stemness in the case of CSCs (12). Thereby, blocking IL6/IL6R by inhibitors or mAbs such as tocilizumab can present itself as a double-edged sword in this case (12, 174). In addition, inhibiting IL6 inhibits MFG-E8, a supporter of the CSC phenotype (194). In the same way, targeting TGFβ might also provide a positive response (12). Another intriguing target is merTK (myeloid-epithelial-reproductive tyrosine kinase), a tyrosine kinase receptor found in various cancers as well as TAMs. However, its activities in both cases are contextual (247, 248). In TAMs, merTK attaches to the “eat-me” signal phosphatidylserine on apoptotic cells, causing a process known as “efferocytosis”. This activation of merTK in TAMs blocks pro-inflammatory signaling and shifts macrophages to the M2 phenotype, as well as increasing the production of immunosuppressive cytokines, further impairing the anti-tumor immune response (248, 249). MerTK is shown to be overexpressed in cancer cells and is commonly linked to cell survival, proliferation, metastasis, chemoresistance, and PDL1 expression (247, 248). merTK signaling is also involved in CSC maintenance in glioblastoma multiforme (250). As a result, inhibiting merTK would have a bidirectional effect on CSC and TAMs. UNC2025 is a small-molecule inhibitor, has proven effective against cancer by inducing apoptosis and reducing colony-forming capability in lung cancer and leukemia models (251, 252). It may also reduce CSC number by curbing M2 phenotypic bias and favoring M1 flipping (**Figure 4**). Leukemia stem cells may employ the help of MDSCs differentiating into TAMs for their survival, and therefore, impairing this crosstalk could deplete both subsets (253). Another study made an intriguing observation where macrophages activated by Amphotericin B hampered the sphere formation capacity in glioma (254).

Another interesting avenue for therapeutic strategy is inhibiting the CSC-led M2 polarization and pushing towards M1 activation. However, favoring M1 is not enough; reducing the expression of CD47 on CSCs, providing an opsonizing agent, and targeting CSCs at the same time are crucial for effective cancer treatment (6). As seen above, CD47 antibodies have shown effective results in the phagocytosis pathway (6, 12). Additionally, targeting CSCs by drugs such as metformin induced epigenetic changes, making CSCs susceptible to chemotherapy (255). Our lab findings also reported that pre-treatment of CSCs with aspirin sensitizes them towards chemo-treatment as well (163). An activator of CSC, the WNT pathway, when downregulated along with paclitaxel, significantly reduced the CSC content and tumor growth (12, 256) (**Figure 4**).

## 9 Therapy through modulation of the TME by integrative/alternative/traditional medicines

Besides the mainstream therapeutic interventions, as discussed above, complementary and alternative medicines (CAMs) are also extensively studied for their therapeutic value. However, their potential use in cancer therapy remains underappreciated. Dietary treatments, herbal medicinal products, homeopathic remedies, and numerous food supplements are currently being investigated as “cancer cures” (257). While these treatment regimens show promise, compelling data for their effectiveness are still limited.

As previously discussed, a substantial body of evidence supports tumor immunotherapy as a vital cancer treatment option. Considering the importance and significance of this therapeutic approach, basic science discoveries elucidating the molecular and cellular biology of immune cells such as T cells, macrophages, B cells, NK cells, Tregs, MDSCs, and their secreted inflammatory cytokines and/or chemokines have led to postulate new strategies in this field, such as ICI therapies, adoptive T-cell transfer therapy, and cancer vaccinology (258). To that end, the search for novel potential modulators for immunotherapy, derived from natural products, has gained prominence as a global research priority. Studies focused primarily on natural products such as polyphenols (e.g., curcumin and resveratrol), cardiotonic steroids (e.g., bufalin and digoxin), terpenoids (e.g., paclitaxel and artemisinins), and polysaccharide extracts (e.g., lentinan), exercising the anti-tumor role of the immune system, have received traction (259).

Ginger, a popular anti-inflammatory herb, has been shown to inhibit macrophage activation and function (260). It is used as an anti-inflammatory agent in conditions like rheumatoid arthritis (261). Kim et al. reported ginger’s suppressive effects on reactive oxygen and nitrogen species production and expression of inducible pro-inflammatory genes (262), which are further responsible for cancer prevention. Moreover, ginseng-derived nanoparticles (GDNPs) were isolated and characterized as an immunopotentiator for modifying M2-polarized macrophages (263). Increased melanoma cell apoptosis was caused by GDNPs’ promotion of the transition from M2 to M1 phenotype and production of total reactive oxygen species. TLR-4 and MyD88-mediated signaling were revealed to be required for GDNP-induced M1 polarization (263). The diverse pleiotropic activities of the natural polyphenol curcumin appear to be involved in multiple cell-signaling pathways at various levels of tumorigenesis (264). Curcumin, a potent scavenger of reactive oxygen species (265), not only reduces systemic toxicity in tumor-bearing animals, but is also implicated in its anti-carcinogenic (266), pro-apoptotic (267), anti-angiogenic (268), anti-metastatic (269), immunomodulatory (270), and antioxidant activities (271). In addition to this, curcumin effectively impairs migration in breast CSCs by impeding EMT (272).

Homoeopathy, a CAM component, is acknowledged as one of the safest and most cost-effective therapeutic modalities on a global scale (273, 274). The homeopathic medication, *Phenacetinum*, inhibits the migration and recruitment of endothelial cells, leading to an imbalance of pro-tumoral macrophages, and leads to structural malformation of the vascular network *in vitro* and *in vivo*. This observation explains the extended survival time of B16F1 melanoma-induced C57BL/6 mice and reduced metastasis in a B16F10-induced model at low dilution (275). Reportedly, *Zincum metallicum* 6c changed the phenotype of macrophages from high to low ROS production whereas *Zincum metallicum* 5c enhanced CD54 expression in macrophages (276). The Canova Method (CM), which consists of homeopathic remedies *Aconitum napellus*, *Arsenicum album* (arsenic trioxide), *Bryonia alba*, *Lachesis* muta venom, and *Thuya occidentalis*, has been indicated for the treatment of cancer and pathologies involving a compromised immune system, such as AIDS (277). CM stimulates the immune system by activating macrophages that in turn stimulate lymphocytes to increase their cytotoxic activity in response to tumor growth without inducing chromosomal abnormalities or genotoxicity (277). Active treatment with CM has been reported to result in a delay in the development and a reduction in the size of malignant tumors, as well as an increase in the infiltration of lymphoid cells, granulation tissue, and fibrosis around the tumor, as compared to control (278). *Calcarea carbonica* also induces apoptosis in tumor cells via an immunomodulatory circuit (279). Notably, the equilibrium state of immunosuppressive and immunostimulatory signals in the inflammatory TME is crucial for tumor suppression.

Taking advantage of CAMs in conjunction with immunotherapeutic methods could add to the efficacy of cancer treatment. Consequently, this article aims to emphasize a promising and futuristic immunotherapy approach alone or in combination with conventional or alternative modalities that deploy the immune system as a tool for cancer treatment. The challenge for the future is to collect credible evidence and discern which approaches yield a greater net benefit.

## 10 Discussion

This review looks at how TAMs, which are prominent invading immune cells, define cancer and the immunological landscape of TME. Initially, during the elimination phase, anti-tumor M1 macrophages are attracted and polarized at the tumor site by inflammatory “call” signals, which aid in the active eradication of cancer cells by various methods at their disposal. However, neoplastic cells utilize a variety of immunomodulatory chemokines and cytokines to either recruit or transform the inflammatory subtype into the pro-tumor one. TRMs that come in contact with cancer cells, on the other hand, suffer the same fate, making the TAM proportion mostly of M2 phenotype. This deft management is accomplished not only by releasing soluble molecules, but also by physiologically modifying the TME, favoring hypoxia, and persuading other immune cells to push towards tumor-promoting TAMs. Because the M2 phenotype is more dynamic, it is further subdivided into M2a, M2b, M2c, and M2d, each with a unique combination of cytokine and chemokine profiles. Despite their diversity, these subtypes all have the same purpose: to maintain the pro-tumor environment. M2-like macrophages, in aggregate, return the favor to cancer by supporting numerous aspects of cancer progression such as proliferation, angiogenesis, EMT, and maintaining an immunosuppressive environment (2). These tumor-promoting interactions are the result of a collaborative effort by other TME members as well as TAMs. TAMs and fibroblasts, for example, engage in a three-way conversation with cancer to aid one another (138). TAMs also help other immunosuppressive cells to retain cancer while blocking anti-cancer immune subsets by changing their functions or expressing a large amount of checkpoint molecules on themselves. TAMs express checkpoint inhibitors such as PDL2, B7S1, SIRPα, galectin-9, and VISTA (V-domain Ig-containing suppressor of T-cell activation) in addition to the previously discussed PDL1 and CTLA4 ligands. Surprisingly, PDL1 expression on TAMs rather than on tumor cells was associated with survival in HCC patients (280, 281). While checkpoint molecules such as PDL1 and SIRPα are abundantly present in M2-like TAMs, PDL1 inhibits the proliferation of anti-tumor immune subtypes, while SIRPα interacts with tumor cells, affecting their phagocytic capability (282). TAMs, interestingly, are also credited with increasing the CSC pool. CSCs, the “root cause” of the disease, promote this immunosuppressive type of macrophage, which may explain immune evasion by this small group of cells during tumor initiation, metastasis, and relapse. The tolerogenic environment established is a perfect combination for poor prognosis and early death. A high M2/M1 ratio and higher TAM infiltration have been linked to unfavorable clinical outcomes in patients despite treatment with various anti-cancer drugs (280).

Therapeutic strategies are thus created to combat TAMs by either reducing their numbers by limiting their infiltration in TME, transforming their phenotype from M2 to M1, boosting their phagocytic capacity, or limiting their immunosuppressive strategies (2). However, by restricting the immunological arm, these strategies can sensitize the tumor but do not solve the problem. High expression of merTK and CD47, for example, in CSCs and cancer cells would continue to promote their preservation while evading immune cell-mediated destruction (187, 250). Therapy efforts would be ineffective unless the immune and malignant compartments were addressed together. As a result, treatments such as chemotherapy and other drugs that target cancer cells and sensitize CSCs, in combination with immunotherapy, may increase patients’ odds of relapse-free survival. In the mouse model, for example, a PDL1 blocker (atezolizumab) paired with a TGFβ inhibitor resulted in substantial anti-tumor immune cell infiltration (283), whereas the combination of atezolizumab and nab-paclitaxel decreased tumors and extended survival of patients with metastatic triple-negative breast cancer (NCT02425891) (284). However, employing ICI therapy has limitations; anti-PD1 antibodies are sometimes hijacked by the Fcγ receptor on macrophages, making the macrophages resistant to such treatments and even leading to lung cancer hyper-progression (280).

## 11 Conclusion and future directives

From the numerous modalities available to disarm cancer–TAM crosstalk, choosing the best way forward requires considering the entire picture of the human body. For instance, depletion and avoiding TAM recruitment may hamper the homeostatic immune balance of the tumor and of the body, which may, in turn, lead to other predicaments. Having an immunogenic environment is efficacious against tumors, but the cancer cleverly turns it into a tolerogenic one. Therefore, reverting to an immunogenic anti-tumor offensive condition seems like a fitting way to go ahead, i.e., reprogramming M2 to M1 type. However, as discussed earlier, switching phenotypes comes with its drawbacks, such as the M1 phenotype notwithstanding for a longer period or M1 ending up helping cancer progression by CSC growth. The CAR-M technique may be the next-gen recipe for macrophages to hold on to their M1 phenotype (6). Additionally, targeting CSCs at the same time, directly and by impairing their phagocytic escape route, is of utmost importance to root out future relapses and to curb M1-promoted CSCs. The development of animal model systems that mimic the human system to the closest degree would help to test out the best possible therapeutic intervention in the smallest possible time. Recently developed spatial transcriptomics allow us to get a better view of the activated pathways focusing not only on TAMs but also on the other orchestrating cells in the TME. Owing to the constant developing heterogeneity inside TME, single-cell omics offer snapshots at any given time frame of the ongoing changes to better understand our therapy strategy and targets. Combinatorial immunotherapy with precise targeting of cancer cells and CSCs may pave the way for a future with much fewer deaths related to cancer. Advances in immunobiology and CSC biology enhance the viability of immunotherapy alone and/or in combination with conventional and alternative treatments. For instance, an alluring prospect is employing CAM modality, not only due to its safe nature but also because of its total targeting capability against the immune arm and cancer at the same time.

## Author contributions

GS: Conceptualization, Funding acquisition, Project administration, Supervision, Writing – review & editing. UB: Data curation, Formal Analysis, Investigation, Writing – original draft. TS: Data curation, Writing – original draft, Writing – review & editing. SM: Visualization, Writing – review & editing. SC: Validation, Writing – review & editing. AD: Data curation, Writing – review & editing. SD: Data curation, Writing – review & editing. DN: Resources, Validation, Writing – review & editing. SK: Funding acquisition, Project administration, Resources, Writing – review & editing. TD: Formal Analysis, Investigation, Supervision, Validation, Writing – review & editing.

## Glossary

**Table:**

ADCC	Antibody-dependent cell-mediated cytotoxicity
CAR-M	Chimeric antigen receptor-expressing macrophages
CCL/R	Chemokine ligand/receptor
CD	Cluster of differentiation
CSC	Cancer stem cells
CAML	Cancer-associated macrophage-like cells
CSF	Colony-stimulating factor
CTL	Cytotoxic T cell
CTLA4	Cytotoxic T-lymphocyte-associated antigen 4
CXCL/R	Chemokine (C-X-C motif) ligand/receptor
DC	Dendritic cell
ECM	Extracellular matrix
EGF	Epidermal growth factor
EMT	Epithelial–mesenchymal transition
FGF	Fibroblast growth factor
HIF	Hypoxia-induced transcription factor
ICI	Immune checkpoint inhibitors
IFN	Interferon
IL	Interleukin
LPS	Lipopolysaccharide
MDSC	Myeloid-derived suppressor cell
merTK	Myeloid-epithelial-reproductive tyrosine kinase
MHC	Major histocompatibility complex
MMP	Matrix metalloproteinases
NF*κ*B	Nuclear factor kappa B
NK	Natural killer cell
NOS	Nitric oxide synthase
NSCC	Non-stem cancer cell
PD1/PDL1	Programmed cell death protein 1/ ligand 1
PDGF	Platelet-derived growth factor
TAM	Tumor-associated macrophage
TGFβ	Transforming growth factor-β
Th	T-helper cell
TLR	Toll-like receptor
TME	Tumor microenvironment
TNFα	Tumor necrosis factor α
Treg	T-regulatory cell
VEGF	Vascular endothelial growth factor

## References

[1] Cancer. Available at: https://www.who.int/news-room/fact-sheets/detail/cancer (Accessed August 20, 2023).

[2] Tan Y Wang M Zhang Y Ge S Zhong F Xia G. Tumor-associated macrophages: A potential target for cancer therapy. Front Oncol (2021) 11:693517. doi: 10.3389/fonc.2021.693517 34178692 PMC8222665

[3] Genard G Lucas S Michiels C. Reprogramming of tumor-associated macrophages with anticancer therapies: radiotherapy versus chemo- and immunotherapies. Front Immunol (2017) 8:828. doi: 10.3389/fimmu.2017.00828 28769933 PMC5509958

[4] Hanahan D Weinberg RA. Hallmarks of cancer: the next generation. Cell (2011) 144:646–74. doi: 10.1016/j.cell.2011.02.013 21376230

[5] Beatty GL Gladney WL. Immune escape mechanisms as a guide for cancer immunotherapy. Clin Cancer Res Off J Am Assoc Cancer Res (2015) 21:687–92. doi: 10.1158/1078-0432.CCR-14-1860 PMC4334715 25501578

[6] Anderson NR Minutolo NG Gill S Klichinsky M. Macrophage-based approaches for cancer immunotherapy. Cancer Res (2021) 81:1201–8. doi: 10.1158/0008-5472.CAN-20-2990 33203697

[7] Hao N-B Lü M-H Fan Y-H Cao Y-L Zhang Z-R Yang S-M. Macrophages in tumor microenvironments and the progression of tumors. Clin Dev Immunol (2012) 2012:948098. doi: 10.1155/2012/948098 22778768 PMC3385963

[8] Cotechini T Atallah A Grossman A. Tissue-resident and recruited macrophages in primary tumor and metastatic microenvironments: potential targets in cancer therapy. Cells (2021) 10:960. doi: 10.3390/cells10040960 33924237 PMC8074766

[9] Boutilier AJ Elsawa SF. Macrophage polarization states in the tumor microenvironment. Int J Mol Sci (2021) 22:6995. doi: 10.3390/ijms22136995 34209703 PMC8268869

[10] Bożyk A Wojas-Krawczyk K Krawczyk P Milanowski J. Tumor microenvironment—A short review of cellular and interaction diversity. Biology (2022) 11:929. doi: 10.3390/biology11060929 35741450 PMC9220289

[11] Li M He L Zhu J Zhang P Liang S. Targeting tumor-associated macrophages for cancer treatment. Cell Biosci (2022) 12:85. doi: 10.1186/s13578-022-00823-5 35672862 PMC9172100

[12] Allavena P Digifico E Belgiovine C. Macrophages and cancer stem cells: a malevolent alliance. Mol Med (2021) 27:121. doi: 10.1186/s10020-021-00383-3 34583655 PMC8480058

[13] Raggi C Mousa HS Correnti M Sica A Invernizzi P. Cancer stem cells and tumor-associated macrophages: a roadmap for multitargeting strategies. Oncogene (2016) 35:671–82. doi: 10.1038/onc.2015.132 25961921

[14] Sarkar T Dhar S Sa G. Tumor-infiltrating T-regulatory cells adapt to altered metabolism to promote tumor-immune escape. Curr Res Immunol (2021) 2:132–41. doi: 10.1016/j.crimmu.2021.08.002 PMC9040151 35492399

[15] Truffi M Sorrentino L Corsi F. Fibroblasts in the tumor microenvironment. Adv Exp Med Biol (2020) 1234:15–29. doi: 10.1007/978-3-030-37184-5_2 32040852

[16] Cendrowicz E Sas Z Bremer E Rygiel TP. The role of macrophages in cancer development and therapy. Cancers (2021) 13:1946. doi: 10.3390/cancers13081946 33919517 PMC8073377

[17] Mantovani A Allavena P Marchesi F Garlanda C. Macrophages as tools and targets in cancer therapy. Nat Rev Drug Discov (2022) 21:799–820. doi: 10.1038/s41573-022-00520-5 35974096 PMC9380983

[18] Zhu Y Herndon JM Sojka DK Kim K-W Knolhoff BL Zuo C. Tissue-resident macrophages in pancreatic ductal adenocarcinoma originate from embryonic hematopoiesis and promote tumor progression. Immunity (2017) 47:323–338.e6. doi: 10.1016/j.immuni.2017.07.014 28813661 PMC5578409

[19] Christofides A Strauss L Yeo A Cao C Charest A Boussiotis VA. The complex role of tumor-infiltrating macrophages. Nat Immunol (2022) 23:1148–56. doi: 10.1038/s41590-022-01267-2 PMC10754321 35879449

[20] Etzerodt A Moulin M Doktor TK Delfini M Mossadegh-Keller N Bajenoff M. Tissue-resident macrophages in omentum promote metastatic spread of ovarian cancer. J Exp Med (2020) 217. doi: 10.1084/jem.20191869 PMC7144521 31951251

[21] Müller A Brandenburg S Turkowski K Müller S Vajkoczy P. Resident microglia, and not peripheral macrophages, are the main source of brain tumor mononuclear cells. Int J Cancer (2015) 137:278–88. doi: 10.1002/ijc.29379 25477239

[22] Gutmann DH Kettenmann H. Microglia/brain macrophages as central drivers of brain tumor pathobiology. Neuron (2019) 104:442–9. doi: 10.1016/j.neuron.2019.08.028 PMC7288606 31697921

[23] Bayik D Lathia JD. Cancer stem cell–immune cell crosstalk in tumour progression. Nat Rev Cancer (2021) 21:526–36. doi: 10.1038/s41568-021-00366-w PMC8740903 34103704

[24] Prehn RT. The immune reaction as a stimulator of tumor growth. Science (1972) 176:170–1. doi: 10.1126/science.176.4031.170 5014438

[25] Wynn TA Chawla A Pollard JW. Macrophage biology in development, homeostasis and disease. Nature (2013) 496:445–55. doi: 10.1038/nature12034 PMC3725458 23619691

[26] Sutton TL Patel RK Anderson AN Bowden SG Whalen R Giske NR. Circulating cells with macrophage-like characteristics in cancer: the importance of circulating neoplastic-immune hybrid cells in cancer. Cancers (2022) 14:3871. doi: 10.3390/cancers14163871 36010865 PMC9405966

[27] Adams DL Martin SS Alpaugh RK Charpentier M Tsai S Bergan RC. Circulating giant macrophages as a potential biomarker of solid tumors. Proc Natl Acad Sci (2014) 111:3514–9. doi: 10.1073/pnas.1320198111 PMC3948254 24550495

[28] Augustyn A Adams DL He J Qiao Y Verma V Liao Z. Giant circulating cancer-associated macrophage-like cells are associated with disease recurrence and survival in non–small-cell lung cancer treated with chemoradiation and atezolizumab. Clin Lung Cancer (2021) 22:e451–65. doi: 10.1016/j.cllc.2020.06.016 32798130

[29] Moran JA Adams DL Edelman MJ Lopez P He J Qiao Y. Monitoring PD-L1 expression on circulating tumor–associated cells in recurrent metastatic non–small-cell lung carcinoma predicts response to immunotherapy with radiation therapy. JCO Precis Oncol (2022) 6:e2200457. doi: 10.1200/PO.22.00457 36516370 PMC10166406

[30] Sindrilaru A Peters T Wieschalka S Baican C Baican A Peter H. An unrestrained proinflammatory M1 macrophage population induced by iron impairs wound healing in humans and mice. J Clin Invest (2011) 121:985–97. doi: 10.1172/JCI44490 PMC3049372 21317534

[31] Orecchioni M Ghosheh Y Pramod AB Ley K. Macrophage Polarization: Different Gene Signatures in M1(LPS+) vs. Classically and M2(LPS-) vs. Alternatively Activated Macrophages. Front Immunol (2019) 10:1084. doi: 10.3389/fimmu.2019.01084 31178859 PMC6543837

[32] Stöger JL Gijbels MJJ van der Velden S Manca M van der Loos CM Biessen EAL. Distribution of macrophage polarization markers in human atherosclerosis. Atherosclerosis (2012) 225:461–8. doi: 10.1016/j.atherosclerosis.2012.09.013 23078881

[33] Gensel JC Kopper TJ Zhang B Orr MB Bailey WM. Predictive screening of M1 and M2 macrophages reveals the immunomodulatory effectiveness of post spinal cord injury azithromycin treatment. Sci Rep (2017) 7:40144. doi: 10.1038/srep40144 28057928 PMC5216345

[34] Wang Y Zhang Y Wang Z Zhang J Qiao RR Xu M. Optical/MRI dual-modality imaging of M1 macrophage polarization in atherosclerotic plaque with MARCO-targeted upconversion luminescence probe. Biomaterials (2019) 219:119378. doi: 10.1016/j.biomaterials.2019.119378 31382209

[35] Allavena P Sica A Solinas G Porta C Mantovani A. The inflammatory micro-environment in tumor progression: the role of tumor-associated macrophages. Crit Rev Oncol Hematol (2008) 66:1–9. doi: 10.1016/j.critrevonc.2007.07.004 17913510

[36] Wang N Liang H Zen K. Molecular mechanisms that influence the macrophage m1-m2 polarization balance. Front Immunol (2014) 5:614. doi: 10.3389/fimmu.2014.00614 25506346 PMC4246889

[37] Fujiwara N Kobayashi K. Macrophages in inflammation. Curr Drug Targets Inflammation Allergy (2005) 4:281–6. doi: 10.2174/1568010054022024 16101534

[38] Jaguin M Houlbert N Fardel O Lecureur V. Polarization profiles of human M-CSF-generated macrophages and comparison of M1-markers in classically activated macrophages from GM-CSF and M-CSF origin. Cell Immunol (2013) 281:51–61. doi: 10.1016/j.cellimm.2013.01.010 23454681

[39] Abdelaziz MH Abdelwahab SF Wan J Cai W Huixuan W Jianjun C. Alternatively activated macrophages; a double-edged sword in allergic asthma. J Transl Med (2020) 18:58. doi: 10.1186/s12967-020-02251-w 32024540 PMC7003359

[40] Hesketh M Sahin KB West ZE Murray RZ. Macrophage phenotypes regulate scar formation and chronic wound healing. Int J Mol Sci (2017) 18:1545. doi: 10.3390/ijms18071545 28714933 PMC5536033

[41] Rőszer T. Understanding the mysterious M2 macrophage through activation markers and effector mechanisms. Mediators Inflammation (2015) 2015:816460. doi: 10.1155/2015/816460 PMC4452191 26089604

[42] Zhang Q Sioud M. Tumor-associated macrophage subsets: shaping polarization and targeting. Int J Mol Sci (2023) 24:7493. doi: 10.3390/ijms24087493 37108657 PMC10138703

[43] Lin L Chen Y-S Yao Y-D Chen J-Q Chen J-N Huang S-Y. CCL18 from tumor-associated macrophages promotes angiogenesis in breast cancer. Oncotarget (2015) 6:34758–73. doi: 10.18632/oncotarget.5325 PMC4741488 26416449

[44] Little AC Pathanjeli P Wu Z Bao L Goo LE Yates JA. IL-4/IL-13 stimulated macrophages enhance breast cancer invasion *via* rho-GTPase regulation of synergistic VEGF/CCL-18 signaling. Front Oncol (2019) 9:456. doi: 10.3389/fonc.2019.00456 31214501 PMC6554436

[45] Fu C Jiang L Hao S Liu Z Ding S Zhang W. Activation of the IL-4/STAT6 signaling pathway promotes lung cancer progression by increasing M2 myeloid cells. Front Immunol (2019) 10:2638. doi: 10.3389/fimmu.2019.02638 31798581 PMC6863933

[46] Asai A Tsuchimoto Y Ohama H Fukunishi S Tsuda Y Kobayashi M. Host antitumor resistance improved by the macrophage polarization in a chimera model of patients with HCC. Oncoimmunology (2017) 6:e1299301. doi: 10.1080/2162402X.2017.1299301 28507807 PMC5414886

[47] Sironi M Martinez FO D’Ambrosio D Gattorno M Polentarutti N Locati M. Differential regulation of chemokine production by Fcgamma receptor engagement in human monocytes: association of CCL1 with a distinct form of M2 monocyte activation (M2b, Type 2). J Leukoc Biol (2006) 80:342–9. doi: 10.1189/jlb.1005586 16735693

[48] Cao Q Wang N Qi J Gu Z Shen H. Long non−coding RNA−GAS5 acts as a tumor suppressor in bladder transitional cell carcinoma *via* regulation of chemokine (C−C motif) ligand 1 expression. Mol Med Rep (2016) 13:27–34. doi: 10.3892/mmr.2015.4503 26548923 PMC4686088

[49] Das S Sarrou E Podgrabinska S Cassella M Mungamuri SK Feirt N. Tumor cell entry into the lymph node is controlled by CCL1 chemokine expressed by lymph node lymphatic sinuses. J Exp Med (2013) 210:1509–28. doi: 10.1084/jem.20111627 PMC3727324 23878309

[50] Liu Y Ji X Kang N Zhou J Liang X Li J. Tumor necrosis factor α inhibition overcomes immunosuppressive M2b macrophage-induced bevacizumab resistance in triple-negative breast cancer. Cell Death Dis (2020) 11:993. doi: 10.1038/s41419-020-03161-x 33214550 PMC7678839

[51] Wang L-X Zhang S-X Wu H-J Rong X-L Guo J. M2b macrophage polarization and its roles in diseases. J Leukoc Biol (2019) 106:345–58. doi: 10.1002/JLB.3RU1018-378RR PMC7379745 30576000

[52] Hung C-H Chen F-M Lin Y-C Tsai M-L Wang S-L Chen Y-C. Altered monocyte differentiation and macrophage polarization patterns in patients with breast cancer. BMC Cancer (2018) 18:366. doi: 10.1186/s12885-018-4284-y 29614988 PMC5883269

[53] Yuan A Hsiao Y-J Chen H-Y Chen H-W Ho C-C Chen Y-Y. Opposite effects of M1 and M2 macrophage subtypes on lung cancer progression. Sci Rep (2015) 5:14273. doi: 10.1038/srep14273 26399191 PMC4585843

[54] Kim D Koh J Ko JS Kim HY Lee H Chung DH. Ubiquitin E3 ligase pellino-1 inhibits IL-10-mediated M2c polarization of macrophages, thereby suppressing tumor growth. Immune Netw (2019) 19:e32. doi: 10.4110/in.2019.19.e32 31720043 PMC6829073

[55] Jetten N Verbruggen S Gijbels MJ Post MJ De Winther MPJ Donners MMPC. Anti-inflammatory M2, but not pro-inflammatory M1 macrophages promote angiogenesis in *vivo* . Angiogenesis (2014) 17:109–18. doi: 10.1007/s10456-013-9381-6 24013945

[56] Spiller KL Anfang RR Spiller KJ Ng J Nakazawa KR Daulton JW. The role of macrophage phenotype in vascularization of tissue engineering scaffolds. Biomaterials (2014) 35:4477–88. doi: 10.1016/j.biomaterials.2014.02.012 PMC4000280 24589361

[57] Fu X-L Duan W Su C-Y Mao F-Y Lv Y-P Teng Y-S. Interleukin 6 induces M2 macrophage differentiation by STAT3 activation that correlates with gastric cancer progression. Cancer Immunol Immunother CII (2017) 66:1597–608. doi: 10.1007/s00262-017-2052-5 PMC11028627 28828629

[58] Duluc D Delneste Y Tan F Moles M-P Grimaud L Lenoir J. Tumor-associated leukemia inhibitory factor and IL-6 skew monocyte differentiation into tumor-associated macrophage-like cells. Blood (2007) 110:4319–30. doi: 10.1182/blood-2007-02-072587 17848619

[59] Susek KH Karvouni M Alici E Lundqvist A. The role of CXC chemokine receptors 1-4 on immune cells in the tumor microenvironment. Front Immunol (2018) 9:2159. doi: 10.3389/fimmu.2018.02159 30319622 PMC6167945

[60] Ardighieri L Missale F Bugatti M Gatta LB Pezzali I Monti M. Infiltration by CXCL10 secreting macrophages is associated with antitumor immunity and response to therapy in ovarian cancer subtypes. Front Immunol (2021) 12:690201. doi: 10.3389/fimmu.2021.690201 34220848 PMC8253056

[61] Tymoszuk P Charoentong P Hackl H Spilka R Müller-Holzner E Trajanoski Z. High STAT1 mRNA levels but not its tyrosine phosphorylation are associated with macrophage infiltration and bad prognosis in breast cancer. BMC Cancer (2014) 14:257. doi: 10.1186/1471-2407-14-257 24725474 PMC4021106

[62] Liang T Chen J Xu G Zhang Z Xue J Zeng H. STAT1 and CXCL10 involve in M1 macrophage polarization that may affect osteolysis and bone remodeling in extrapulmonary tuberculosis. Gene (2022) 809:146040. doi: 10.1016/j.gene.2021.146040 34710525

[63] Peng C Tu G Wang J Wang Y Wu P Yu L. MLKL signaling regulates macrophage polarization in acute pancreatitis through CXCL10. Cell Death Dis (2023) 14:1–14. doi: 10.1038/s41419-023-05655-w 36828808 PMC9958014

[64] Shang S Yang Y-W Chen F Yu L Shen S-H Li K. TRIB3 reduces CD8+ T cell infiltration and induces immune evasion by repressing the STAT1-CXCL10 axis in colorectal cancer. Sci Transl Med (2022) 14:eabf0992. doi: 10.1126/scitranslmed.abf0992 34985967

[65] Sallusto F Lenig D Mackay CR Lanzavecchia A. Flexible programs of chemokine receptor expression on human polarized T helper 1 and 2 lymphocytes. J Exp Med (1998) 187:875–83. doi: 10.1084/jem.187.6.875 PMC2212187 9500790

[66] Bronger H Singer J Windmüller C Reuning U Zech D Delbridge C. CXCL9 and CXCL10 predict survival and are regulated by cyclooxygenase inhibition in advanced serous ovarian cancer. Br J Cancer (2016) 115:553–63. doi: 10.1038/bjc.2016.172 PMC4997538 27490802

[67] Chheda ZS Sharma RK Jala VR Luster AD Haribabu B. Chemoattractant receptors BLT1 and CXCR3 regulate antitumor immunity by facilitating CD8+ T cell migration into tumors. J Immunol (2016) 197:2016–26. doi: 10.4049/jimmunol.1502376 PMC4992661 27465528

[68] Li CX Ling CC Shao Y Xu A Li XC Ng KT-P. CXCL10/CXCR3 signaling mobilized-regulatory T cells promote liver tumor recurrence after transplantation. J Hepatol (2016) 65:944–52. doi: 10.1016/j.jhep.2016.05.032 27245433

[69] Wennerberg E Kremer V Childs R Lundqvist A. CXCL10-induced migration of adoptively transferred human natural killer cells toward solid tumors causes regression of tumor growth in *vivo* . Cancer Immunol Immunother (2015) 64:225–35. doi: 10.1007/s00262-014-1629-5 PMC11028951 25344904

[70] Yue C Shen S Deng J Priceman SJ Li W Huang A. STAT3 in CD8+ T cells inhibits their tumor accumulation by downregulating CXCR3/CXCL10 axis. Cancer Immunol Res (2015) 3:864–70. doi: 10.1158/2326-6066.CIR-15-0014 PMC4527884 26025380

[71] Mosser DM Edwards JP. Exploring the full spectrum of macrophage activation. Nat Rev Immunol (2008) 8:958–69. doi: 10.1038/nri2448 PMC2724991 19029990

[72] Zhang M He Y Sun X Li Q Wang W Zhao A. A high M1/M2 ratio of tumor-associated macrophages is associated with extended survival in ovarian cancer patients. J Ovarian Res (2014) 7:19. doi: 10.1186/1757-2215-7-19 24507759 PMC3939626

[73] Ma J Liu L Che G Yu N Dai F You Z. The M1 form of tumor-associated macrophages in non-small cell lung cancer is positively associated with survival time. BMC Cancer (2010) 10:112. doi: 10.1186/1471-2407-10-112 20338029 PMC2851690

[74] Jackute J Zemaitis M Pranys D Sitkauskiene B Miliauskas S Vaitkiene S. Distribution of M1 and M2 macrophages in tumor islets and stroma in relation to prognosis of non-small cell lung cancer. BMC Immunol (2018) 19:3. doi: 10.1186/s12865-018-0241-4 29361917 PMC5781310

[75] Edin S Wikberg ML Oldenborg P-A Palmqvist R. Macrophages: Good guys in colorectal cancer. Oncoimmunology (2013) 2:e23038. doi: 10.4161/onci.23038 23524684 PMC3601174

[76] Honkanen TJ Tikkanen A Karihtala P Mäkinen M Väyrynen JP Koivunen JP. Prognostic and predictive role of tumour-associated macrophages in HER2 positive breast cancer. Sci Rep (2019) 9:10961. doi: 10.1038/s41598-019-47375-2 31358801 PMC6662906

[77] Alves AM Diel LF Lamers ML. Macrophages and prognosis of oral squamous cell carcinoma: A systematic review. J Oral Pathol Med Off Publ Int Assoc Oral Pathol Am Acad Oral Pathol (2018) 47:460–7. doi: 10.1111/jop.12643 28940738

[78] Jayasingam SD Citartan M Thang TH Mat Zin AA Ang KC Ch’ng ES. Evaluating the polarization of tumor-associated macrophages into M1 and M2 phenotypes in human cancer tissue: technicalities and challenges in routine clinical practice. Front Oncol (2019) 9:1512. doi: 10.3389/fonc.2019.01512 32039007 PMC6992653

[79] Müller E Christopoulos PF Halder S Lunde A Beraki K Speth M. Toll-like receptor ligands and interferon-γ Synergize for induction of antitumor M1 macrophages. Front Immunol (2017) 8:1383. doi: 10.3389/fimmu.2017.01383 29123526 PMC5662546

[80] Li N Qin J Lan L Zhang H Liu F Wu Z. PTEN inhibits macrophage polarization from M1 to M2 through CCL2 and VEGF-A reduction and NHERF-1 synergism. Cancer Biol Ther (2015) 16:297–306. doi: 10.1080/15384047.2014.1002353 25756512 PMC4622010

[81] Fukao T Koyasu S. PI3K and negative regulation of TLR signaling. Trends Immunol (2003) 24:358–63. doi: 10.1016/S1471-4906(03)00139-X 12860525

[82] Kratochvill F Neale G Haverkamp JM Van de Velde L-A Smith AM Kawauchi D. TNF counterbalances the emergence of M2 tumor macrophages. Cell Rep (2015) 12:1902–14. doi: 10.1016/j.celrep.2015.08.033 PMC4581986 26365184

[83] Klimp AH de Vries EGE Scherphof GL Daemen T. A potential role of macrophage activation in the treatment of cancer. Crit Rev Oncol Hematol (2002) 44:143–61. doi: 10.1016/S1040-8428(01)00203-7 12413632

[84] Pan Y Yu Y Wang X Zhang T. Tumor-associated macrophages in tumor immunity. Front Immunol (2020) 11:583084. doi: 10.3389/fimmu.2020.583084 33365025 PMC7751482

[85] Bernsmeier C van der Merwe S Périanin A. Innate immune cells in cirrhosis. J Hepatol (2020) 73:186–201. doi: 10.1016/j.jhep.2020.03.027 32240716

[86] Garbán HJ Bonavida B. Nitric oxide sensitizes ovarian tumor cells to Fas-induced apoptosis. Gynecol Oncol (1999) 73:257–64. doi: 10.1006/gyno.1999.5374 10329044

[87] Duluc D Corvaisier M Blanchard S Catala L Descamps P Gamelin E. Interferon-γ reverses the immunosuppressive and protumoral properties and prevents the generation of human tumor-associated macrophages. Int J Cancer (2009) 125:367–73. doi: 10.1002/ijc.24401 19378341

[88] Chen S Lai SWT Brown CE Feng M. Harnessing and enhancing macrophage phagocytosis for cancer therapy. Front Immunol (2021) 12:635173. doi: 10.3389/fimmu.2021.635173 33790906 PMC8006289

[89] Bruns H Büttner M Fabri M Mougiakakos D Bittenbring JT Hoffmann MH. Vitamin D-dependent induction of cathelicidin in human macrophages results in cytotoxicity against high-grade B cell lymphoma. Sci Transl Med (2015) 7:282ra47. doi: 10.1126/scitranslmed.aaa3230 25855493

[90] Ma P-F Gao C-C Yi J Zhao J-L Liang S-Q Zhao Y. Cytotherapy with M1-polarized macrophages ameliorates liver fibrosis by modulating immune microenvironment in mice. J Hepatol (2017) 67:770–9. doi: 10.1016/j.jhep.2017.05.022 28596109

[91] Sica A Mantovani A. Macrophage plasticity and polarization: in *vivo* veritas. J Clin Invest (2012) 122:787–95. doi: 10.1172/JCI59643 PMC3287223 22378047

[92] Yin C Han Q Xu D Zheng B Zhao X Zhang J. SALL4-mediated upregulation of exosomal miR-146a-5p drives T-cell exhaustion by M2 tumor-associated macrophages in HCC. Oncoimmunology (2019) 8:1601479. doi: 10.1080/2162402X.2019.1601479 31143524 PMC6527304

[93] Bohn T Rapp S Luther N Klein M Bruehl T-J Kojima N. Tumor immunoevasion *via* acidosis-dependent induction of regulatory tumor-associated macrophages. Nat Immunol (2018) 19:1319–29. doi: 10.1038/s41590-018-0226-8 30397348

[94] Colegio OR Chu N-Q Szabo AL Chu T Rhebergen AM Jairam V. Functional polarization of tumour-associated macrophages by tumour-derived lactic acid. Nature (2014) 513:559–63. doi: 10.1038/nature13490 PMC4301845 25043024

[95] Goossens P Rodriguez-Vita J Etzerodt A Masse M Rastoin O Gouirand V. Membrane cholesterol efflux drives tumor-associated macrophage reprogramming and tumor progression. Cell Metab (2019) 29:1376–1389.e4. doi: 10.1016/j.cmet.2019.02.016 30930171

[96] Palmieri EM Menga A Martín-Pérez R Quinto A Riera-Domingo C De Tullio G. Pharmacologic or genetic targeting of glutamine synthetase skews macrophages toward an M1-like phenotype and inhibits tumor metastasis. Cell Rep (2017) 20:1654–66. doi: 10.1016/j.celrep.2017.07.054 PMC5575233 28813676

[97] Murdoch C Muthana M Lewis CE. Hypoxia regulates macrophage functions in inflammation. J Immunol Baltim Md 1950 (2005) 175:6257–63. doi: 10.4049/jimmunol.175.10.6257 16272275

[98] Huber R Meier B Otsuka A Fenini G Satoh T Gehrke S. Tumour hypoxia promotes melanoma growth and metastasis *via* High Mobility Group Box-1 and M2-like macrophages. Sci Rep (2016) 6:29914. doi: 10.1038/srep29914 27426915 PMC4947927

[99] Liu C Chikina M Deshpande R Menk AV Wang T Tabib T. Treg cells promote the SREBP1-dependent metabolic fitness of tumor-promoting macrophages *via* repression of CD8+ T cell-derived interferon-γ. Immunity (2019) 51:381–397.e6. doi: 10.1016/j.immuni.2019.06.017 31350177 PMC6703933

[100] Kumar V Cheng P Condamine T Mony S Languino LR McCaffrey JC. CD45 phosphatase inhibits STAT3 transcription factor activity in myeloid cells and promotes tumor-associated macrophage differentiation. Immunity (2016) 44:303–15. doi: 10.1016/j.immuni.2016.01.014 PMC4759655 26885857

[101] Roghanian A Fraser C Kleyman M Chen J. B cells promote pancreatic tumorigenesis. Cancer Discovery (2016) 6:230–2. doi: 10.1158/2159-8290.CD-16-0100 26951836

[102] Kawane K Fukuyama H Kondoh G Takeda J Ohsawa Y Uchiyama Y. Requirement of DNase II for definitive erythropoiesis in the mouse fetal liver. Science (2001) 292:1546–9. doi: 10.1126/science.292.5521.1546 11375492

[103] Chong BF Tseng L-C Hosler GA Teske NM Zhang S Karp DR. A subset of CD163+ macrophages displays mixed polarizations in discoid lupus skin. Arthritis Res Ther (2015) 17:324. doi: 10.1186/s13075-015-0839-3 26568320 PMC4644297

[104] Lewis CE Pollard JW. Distinct role of macrophages in different tumor microenvironments. Cancer Res (2006) 66:605–12. doi: 10.1158/0008-5472.CAN-05-4005 16423985

[105] Polverini PJ Leibovich SJ. Effect of macrophage depletion on growth and neovascularization of hamster buccal pouch carcinomas. J Oral Pathol (1987) 16:436–41. doi: 10.1111/j.1600-0714.1987.tb00714.x 2448437

[106] Mitsudomi T Yatabe Y. Epidermal growth factor receptor in relation to tumor development: EGFR gene and cancer. FEBS J (2010) 277:301–8. doi: 10.1111/j.1742-4658.2009.07448.x 19922469

[107] Huynh LK Hipolito CJ ten Dijke P. A perspective on the development of TGF-β Inhibitors for cancer treatment. Biomolecules (2019) 9:743. doi: 10.3390/biom9110743 31744193 PMC6921009

[108] Liu C Zhang W Wang J Si T Xing W. Tumor-associated macrophage-derived transforming growth factor-β promotes colorectal cancer progression through HIF1-TRIB3 signaling. Cancer Sci (2021) 112:4198–207. doi: 10.1111/cas.15101 PMC8486199 34375482

[109] Guruvayoorappan C. Tumor versus tumor-associated macrophages: how hot is the link? Integr Cancer Ther (2008) 7:90–5. doi: 10.1177/1534735408319060 18550889

[110] Chen P Huang Y Bong R Ding Y Song N Wang X. Tumor-associated macrophages promote angiogenesis and melanoma growth *via* adrenomedullin in a paracrine and autocrine manner. Clin Cancer Res Off J Am Assoc Cancer Res (2011) 17:7230–9. doi: 10.1158/1078-0432.CCR-11-1354 21994414

[111] Folkman J. Tumor angiogenesis: therapeutic implications. N Engl J Med (1971) 285:1182–6. doi: 10.1056/NEJM197111182852108 4938153

[112] Zeisberger SM Odermatt B Marty C Zehnder-Fjällman AHM Ballmer-Hofer K Schwendener RA. Clodronate-liposome-mediated depletion of tumour-associated macrophages: a new and highly effective antiangiogenic therapy approach. Br J Cancer (2006) 95:272–81. doi: 10.1038/sj.bjc.6603240 PMC2360657 16832418

[113] Lugano R Ramachandran M Dimberg A. Tumor angiogenesis: causes, consequences, challenges and opportunities. Cell Mol Life Sci (2020) 77:1745–70. doi: 10.1007/s00018-019-03351-7 PMC7190605 31690961

[114] Solinas G Germano G Mantovani A Allavena P. Tumor-associated macrophages (TAM) as major players of the cancer-related inflammation. J Leukoc Biol (2009) 86:1065–73. doi: 10.1189/jlb.0609385 19741157

[115] Siveen KS Kuttan G. Role of macrophages in tumour progression. Immunol Lett (2009) 123:97–102. doi: 10.1016/j.imlet.2009.02.011 19428556

[116] Vérollet C Charrière GM Labrousse A Cougoule C Le Cabec V Maridonneau-Parini I. Extracellular proteolysis in macrophage migration: losing grip for a breakthrough. Eur J Immunol (2011) 41:2805–13. doi: 10.1002/eji.201141538 21953638

[117] Schioppa T Uranchimeg B Saccani A Biswas SK Doni A Rapisarda A. Regulation of the chemokine receptor CXCR4 by hypoxia. J Exp Med (2003) 198:1391–402. doi: 10.1084/jem.20030267 PMC2194248 14597738

[118] Bai R Li Y Jian L Yang Y Zhao L Wei M. The hypoxia-driven crosstalk between tumor and tumor-associated macrophages: mechanisms and clinical treatment strategies. Mol Cancer (2022) 21:177. doi: 10.1186/s12943-022-01645-2 36071472 PMC9454207

[119] Liu Q Zhao E Geng B Gao S Yu H He X. Tumor-associated macrophage-derived exosomes transmitting miR-193a-5p promote the progression of renal cell carcinoma *via* TIMP2-dependent vasculogenic mimicry. Cell Death Dis (2022) 13:1–14. doi: 10.1038/s41419-022-04814-9 PMC9021253 35443741

[120] Schoppmann SF Birner P Stöckl J Kalt R Ullrich R Caucig C. Tumor-associated macrophages express lymphatic endothelial growth factors and are related to peritumoral lymphangiogenesis. Am J Pathol (2002) 161:947–56. doi: 10.1016/S0002-9440(10)64255-1 PMC1867252 12213723

[121] Kerjaschki D. The crucial role of macrophages in lymphangiogenesis. J Clin Invest (2005) 115:2316–9. doi: 10.1172/JCI26354 PMC1193892 16138185

[122] Rhim AD Mirek ET Aiello NM Maitra A Bailey JM McAllister F. EMT and dissemination precede pancreatic tumor formation. Cell (2012) 148:349–61. doi: 10.1016/j.cell.2011.11.025 PMC3266542 22265420

[123] Gorelik E Wiltrout RH Brunda MJ Holden HT Herberman RB. Augmentation of metastasis formation by thioglycollate-elicited macrophages. Int J Cancer (1982) 29:575–81. doi: 10.1002/ijc.2910290514 7095902

[124] Coffelt SB Hughes R Lewis CE. Tumor-associated macrophages: Effectors of angiogenesis and tumor progression. Biochim Biophys Acta BBA - Rev Cancer (2009) 1796:11–8. doi: 10.1016/j.bbcan.2009.02.004 19269310

[125] Hagemann T Robinson SC Schulz M Trümper L Balkwill FR Binder C. Enhanced invasiveness of breast cancer cell lines upon co-cultivation with macrophages is due to TNF-α dependent up-regulation of matrix metalloproteases. Carcinogenesis (2004) 25:1543–9. doi: 10.1093/carcin/bgh146 15044327

[126] Lynch CC Hikosaka A Acuff HB Martin MD Kawai N Singh RK. MMP-7 promotes prostate cancer-induced osteolysis *via* the solubilization of RANKL. Cancer Cell (2005) 7:485–96. doi: 10.1016/j.ccr.2005.04.013 15894268

[127] Winkler J Abisoye-Ogunniyan A Metcalf KJ Werb Z. Concepts of extracellular matrix remodelling in tumour progression and metastasis. Nat Commun (2020) 11:5120. doi: 10.1038/s41467-020-18794-x 33037194 PMC7547708

[128] Xu J Lamouille S Derynck R. TGF-beta-induced epithelial to mesenchymal transition. Cell Res (2009) 19:156–72. doi: 10.1038/cr.2009.5 PMC4720263 19153598

[129] Su S Liu Q Chen J Chen J Chen F He C. A positive feedback loop between mesenchymal-like cancer cells and macrophages is essential to breast cancer metastasis. Cancer Cell (2014) 25:605–20. doi: 10.1016/j.ccr.2014.03.021 24823638

[130] Zhang J Yan Y Yang Y Wang L Li M Wang J. High infiltration of tumor-associated macrophages influences poor prognosis in human gastric cancer patients, associates with the phenomenon of EMT. Med (Baltimore) (2016) 95:e2636. doi: 10.1097/MD.0000000000002636 PMC4753880 26871785

[131] Madsen DH Jürgensen HJ Siersbæk MS Kuczek DE Grey Cloud L Liu S. Tumor-associated macrophages derived from circulating inflammatory monocytes degrade collagen through cellular uptake. Cell Rep (2017) 21:3662–71. doi: 10.1016/j.celrep.2017.12.011 PMC5753792 29281816

[132] Raskov H Orhan A Gaggar S Gögenur I. Cancer-associated fibroblasts and tumor-associated macrophages in cancer and cancer immunotherapy. Front Oncol (2021) 11:668731. doi: 10.3389/fonc.2021.668731 34094963 PMC8172975

[133] Comito G Giannoni E Segura CP Barcellos-de-Souza P Raspollini MR Baroni G. Cancer-associated fibroblasts and M2-polarized macrophages synergize during prostate carcinoma progression. Oncogene (2014) 33:2423–31. doi: 10.1038/onc.2013.191 23728338

[134] Gok Yavuz B Gunaydin G Gedik ME Kosemehmetoglu K Karakoc D Ozgur F. Cancer associated fibroblasts sculpt tumour microenvironment by recruiting monocytes and inducing immunosuppressive PD-1+ TAMs. Sci Rep (2019) 9:3172. doi: 10.1038/s41598-019-39553-z 30816272 PMC6395633

[135] Tokuda K Morine Y Miyazaki K Yamada S Saito Y Nishi M. The interaction between cancer associated fibroblasts and tumor associated macrophages *via* the osteopontin pathway in the tumor microenvironment of hepatocellular carcinoma. Oncotarget (2021) 12:333–43. doi: 10.18632/oncotarget.27881 PMC7899554 33659044

[136] Lee H-O Mullins SR Franco-Barraza J Valianou M Cukierman E Cheng JD. FAP-overexpressing fibroblasts produce an extracellular matrix that enhances invasive velocity and directionality of pancreatic cancer cells. BMC Cancer (2011) 11:245. doi: 10.1186/1471-2407-11-245 21668992 PMC3141768

[137] Gaggioli C Hooper S Hidalgo-Carcedo C Grosse R Marshall JF Harrington K. Fibroblast-led collective invasion of carcinoma cells with differing roles for RhoGTPases in leading and following cells. Nat Cell Biol (2007) 9:1392–400. doi: 10.1038/ncb1658 18037882

[138] Gunaydin G. CAFs interacting with TAMs in tumor microenvironment to enhance tumorigenesis and immune evasion. Front Oncol (2021) 11:668349. doi: 10.3389/fonc.2021.668349 34336660 PMC8317617

[139] Jiang H Hegde S DeNardo DG. Tumor-associated fibrosis as a regulator of tumor immunity and response to immunotherapy. Cancer Immunol Immunother (2017) 66:1037–48. doi: 10.1007/s00262-017-2003-1 PMC5603233 28451791

[140] Kobayashi N Miyoshi S Mikami T Koyama H Kitazawa M Takeoka M. Hyaluronan deficiency in tumor stroma impairs macrophage trafficking and tumor neovascularization. Cancer Res (2010) 70:7073–83. doi: 10.1158/0008-5472.CAN-09-4687 20823158

[141] Dvorak HF. Tumors: wounds that do not heal. Similarities between tumor stroma generation and wound healing. N Engl J Med (1986) 315:1650–9. doi: 10.1056/NEJM198612253152606 3537791

[142] Liu Y Cao X. Immunosuppressive cells in tumor immune escape and metastasis. J Mol Med Berl Ger (2016) 94:509–22. doi: 10.1007/s00109-015-1376-x 26689709

[143] Mantovani A Sica A. Macrophages, innate immunity and cancer: balance, tolerance, and diversity. Curr Opin Immunol (2010) 22:231–7. doi: 10.1016/j.coi.2010.01.009 20144856

[144] Ito M Minamiya Y Kawai H Saito S Saito H Nakagawa T. Tumor-derived TGFbeta-1 induces dendritic cell apoptosis in the sentinel lymph node. J Immunol Baltim Md 1950 (2006) 176:5637–43. doi: 10.4049/jimmunol.176.9.5637 16622033

[145] Bied M Ho WW Ginhoux F Blériot C. Roles of macrophages in tumor development: a spatiotemporal perspective. Cell Mol Immunol (2023) 20:983–92. doi: 10.1038/s41423-023-01061-6 PMC10468537 37429944

[146] Flavell RA Sanjabi S Wrzesinski SH Licona-Limón P. The polarization of immune cells in the tumour environment by TGFbeta. Nat Rev Immunol (2010) 10:554–67. doi: 10.1038/nri2808 PMC3885992 20616810

[147] Bak SP Alonso A Turk MJ Berwin B. Murine ovarian cancer vascular leukocytes require arginase-1 activity for T cell suppression. Mol Immunol (2008) 46:258–68. doi: 10.1016/j.molimm.2008.08.266 PMC2613193 18824264

[148] Gabrilovich DI Nagaraj S. Myeloid-derived suppressor cells as regulators of the immune system. Nat Rev Immunol (2009) 9:162–74. doi: 10.1038/nri2506 PMC2828349 19197294

[149] Sarkar T Dhar S Chakraborty D Pati S Bose S Panda AK. FOXP3/HAT1 axis controls treg infiltration in the tumor microenvironment by inducing CCR4 expression in breast cancer. Front Immunol (2022) 13:740588. doi: 10.3389/fimmu.2022.740588 35222362 PMC8863663

[150] Anderson KM Czinn SJ Redline RW Blanchard TG. Induction of CTLA-4-mediated anergy contributes to persistent colonization in the murine model of gastric helicobacter pylori infection1. J Immunol (2006) 176:5306–13. doi: 10.4049/jimmunol.176.9.5306 16621997

[151] Zhang H Dai Z Wu W Wang Z Zhang N Zhang L. Regulatory mechanisms of immune checkpoints PD-L1 and CTLA-4 in cancer. J Exp Clin Cancer Res (2021) 40:184. doi: 10.1186/s13046-021-01987-7 34088360 PMC8178863

[152] Jeong H Kim S Hong B-J Lee C-J Kim Y-E Bok S. Tumor-associated macrophages enhance tumor hypoxia and aerobic glycolysis. Cancer Res (2019) 79:795–806. doi: 10.1158/0008-5472.CAN-18-2545 30610087

[153] Chen M-M Xiao X Lao X-M Wei Y Liu R-X Zeng Q-H. Polarization of tissue-resident TFH-like cells in human hepatoma bridges innate monocyte inflammation and M2b macrophage polarization. Cancer Discovery (2016) 6:1182–95. doi: 10.1158/2159-8290.CD-16-0329 27531854

[154] Reya T Morrison SJ Clarke MF Weissman IL. Stem cells, cancer, and cancer stem cells. Nature (2001) 414:105–11. doi: 10.1038/35102167 11689955

[155] Kreso A Dick JE. Evolution of the cancer stem cell model. Cell Stem Cell (2014) 14:275–91. doi: 10.1016/j.stem.2014.02.006 24607403

[156] Celià-Terrassa T Jolly MK. Cancer stem cells and epithelial-to-mesenchymal transition in cancer metastasis. Cold Spring Harb Perspect Med (2020) 10:a036905. doi: 10.1101/cshperspect.a036905 31570380 PMC7328448

[157] Celià-Terrassa T Kang Y. Distinctive properties of metastasis-initiating cells. Genes Dev (2016) 30:892–908. doi: 10.1101/gad.277681.116 27083997 PMC4840296

[158] Kleiner DE Stetler-Stevenson WG. Matrix metalloproteinases and metastasis. Cancer Chemother Pharmacol (1999) 43:S42–51. doi: 10.1007/s002800051097 10357558

[159] Oskarsson T Batlle E Massagué J. Metastatic stem cells: sources, niches, and vital pathways. Cell Stem Cell (2014) 14:306–21. doi: 10.1016/j.stem.2014.02.002 PMC3998185 24607405

[160] Mukherjee S Manna A Bhattacharjee P Mazumdar M Saha S Chakraborty S. Non-migratory tumorigenic intrinsic cancer stem cells ensure breast cancer metastasis by generation of CXCR4+ migrating cancer stem cells. Oncogene (2016) 35:4937–48. doi: 10.1038/onc.2016.26 26923331

[161] Jin L Han B Siegel E Cui Y Giuliano A Cui X. Breast cancer lung metastasis: Molecular biology and therapeutic implications. Cancer Biol Ther (2018) 19:858–68. doi: 10.1080/15384047.2018.1456599 PMC6300341 29580128

[162] Khan P Manna A Saha S Mohanty S Mukherjee S Mazumdar M. Aspirin inhibits epithelial-to-mesenchymal transition and migration of oncogenic K-ras-expressing non-small cell lung carcinoma cells by down-regulating E-cadherin repressor Slug. BMC Cancer (2016) 16:39. doi: 10.1186/s12885-016-2078-7 26810856 PMC4727308

[163] Saha S Mukherjee S Khan P Kajal K Mazumdar M Manna A. Aspirin suppresses the acquisition of chemoresistance in breast cancer by disrupting an NFκB-IL6 signaling axis responsible for the generation of cancer stem cells. Cancer Res (2016) 76:2000–12. doi: 10.1158/0008-5472.CAN-15-1360 26842876

[164] Chen L Yang F Chen S Tai J. Mechanisms on chemotherapy resistance of colorectal cancer stem cells and research progress of reverse transformation: A mini-review. Front Med (2022) 9:995882. doi: 10.3389/fmed.2022.995882 PMC9510709 36172536

[165] Yu Y Ramena G Elble RC. The role of cancer stem cells in relapse of solid tumors. Front Biosci Elite Ed (2012) 4:1528–41. doi: 10.2741/e478 22201973

[166] Chakraborty S Mukherjee S Basak U Pati S Dutta A Dutta S. Immune evasion by cancer stem cells ensures tumor initiation and failure of immunotherapy. Explor Immunol (2023) 3:384–405. doi: 10.37349/ei.2023.00108

[167] Müller L Tunger A Plesca I Wehner R Temme A Westphal D. Bidirectional crosstalk between cancer stem cells and immune cell subsets. Front Immunol (2020) 11:140. doi: 10.3389/fimmu.2020.00140 32117287 PMC7013084

[168] Stein RG Ebert S Schlahsa L Scholz CJ Braun M Hauck P. Cognate nonlytic interactions between CD8+ T cells and breast cancer cells induce cancer stem cell–like properties. Cancer Res (2019) 79:1507–19. doi: 10.1158/0008-5472.CAN-18-0387 30692216

[169] Li K Shi H Zhang B Ou X Ma Q Chen Y. Myeloid-derived suppressor cells as immunosuppressive regulators and therapeutic targets in cancer. Signal Transduct Target Ther (2021) 6:1–25. doi: 10.1038/s41392-021-00670-9 34620838 PMC8497485

[170] Roy D Bose S Pati S Guin A Banerjee K Saha S. GFI1/HDAC1-axis differentially regulates immunosuppressive CD73 in human tumor-associated FOXP3+ Th17 and inflammation-linked Th17 cells. Eur J Immunol (2021) 51:1206–17. doi: 10.1002/eji.202048892 33555624

[171] Zeppernick F Ahmadi R Campos B Dictus C Helmke BM Becker N. Stem cell marker CD133 affects clinical outcome in glioma patients. Clin Cancer Res Off J Am Assoc Cancer Res (2008) 14:123–9. doi: 10.1158/1078-0432.CCR-07-0932 18172261

[172] Yeung TM Gandhi SC Bodmer WF. Hypoxia and lineage specification of cell line-derived colorectal cancer stem cells. Proc Natl Acad Sci (2011) 108:4382–7. doi: 10.1073/pnas.1014519107 PMC3060223 21368208

[173] Lu H Clauser KR Tam WL Fröse J Ye X Eaton EN. A breast cancer stem cell niche supported by juxtacrine signalling from monocytes and macrophages. Nat Cell Biol (2014) 16:1105–17. doi: 10.1038/ncb3041 PMC4296514 25266422

[174] Wan S Zhao E Kryczek I Vatan L Sadovskaya A Ludema G. Tumor-associated macrophages produce interleukin 6 and signal *via* STAT3 to promote expansion of human hepatocellular carcinoma stem cells. Gastroenterology (2014) 147:1393–404. doi: 10.1053/j.gastro.2014.08.039 PMC4253315 25181692

[175] Luo S Yang G Ye P Cao N Chi X Yang W-H. Macrophages are a double-edged sword: molecular crosstalk between tumor-associated macrophages and cancer stem cells. Biomolecules (2022) 12:850. doi: 10.3390/biom12060850 35740975 PMC9221070

[176] Wu A Wei J Kong L-Y Wang Y Priebe W Qiao W. Glioma cancer stem cells induce immunosuppressive macrophages/microglia. Neuro-Oncol (2010) 12:1113–25. doi: 10.1093/neuonc/noq082 PMC3098021 20667896

[177] Wang J Wang X Wang Y Li S Wang X. Krüppel like factor 6 splice variant 1 (KLF6-SV1) overexpression recruits macrophages to participate in lung cancer metastasis by up-regulating TWIST1. Cancer Biol Ther (2019) 20:680–91. doi: 10.1080/15384047.2018.1550570 PMC6605981 30590988

[178] Ji J Wang P Zhou Q Zhu L Zhang H Zhang Y. CCL8 enhances sensitivity of cutaneous squamous cell carcinoma to photodynamic therapy by recruiting M1 macrophages. Photodiagnosis Photodyn Ther (2019) 26:235–43. doi: 10.1016/j.pdpdt.2019.03.014 30902794

[179] Zhang X Chen L Dang W Cao M Xiao J Lv S. CCL8 secreted by tumor-associated macrophages promotes invasion and stemness of glioblastoma cells *via* ERK1/2 signaling. Lab Invest (2020) 100:619–29. doi: 10.1038/s41374-019-0345-3 31748682

[180] De Boeck A Ahn BY D’Mello C Lun X Menon SV Alshehri MM. Glioma-derived IL-33 orchestrates an inflammatory brain tumor microenvironment that accelerates glioma progression. Nat Commun (2020) 11:4997. doi: 10.1038/s41467-020-18569-4 33020472 PMC7536425

[181] Zhang F Li P Liu S Yang M Zeng S Deng J. β-Catenin-CCL2 feedback loop mediates crosstalk between cancer cells and macrophages that regulates breast cancer stem cells. Oncogene (2021) 40:5854–65. doi: 10.1038/s41388-021-01986-0 34345015

[182] Zhuang Y Zhao X Yuan B Zeng Z Chen Y. Blocking the CCL5&ndash;CCR5 axis using maraviroc promotes M1 polarization of macrophages cocultured with irradiated hepatoma cells. J Hepatocell Carcinoma (2021) 8:599–611. doi: 10.2147/JHC.S300165 34178876 PMC8219307

[183] Deng X Zhang P Liang T Deng S Chen X Zhu L. Ovarian cancer stem cells induce the M2 polarization of macrophages through the PPARγ and NF-κB pathways. Int J Mol Med (2015) 36:449–54. doi: 10.3892/ijmm.2015.2230 26035689

[184] Liu Q Wu H Li Y Zhang R Kleeff J Zhang X. Combined blockade of TGf-β1 and GM-CSF improves chemotherapeutic effects for pancreatic cancer by modulating tumor microenvironment. Cancer Immunol Immunother (2020) 69:1477–92. doi: 10.1007/s00262-020-02542-7 PMC11027661 32285172

[185] Gabrusiewicz K Li X Wei J Hashimoto Y Marisetty AL Ott M. Glioblastoma stem cell-derived exosomes induce M2 macrophages and PD-L1 expression on human monocytes. OncoImmunology (2018) 7:e1412909. doi: 10.1080/2162402X.2017.1412909 29632728 PMC5889290

[186] Bayik D Lathia JD. Cancer stem cell-immune cell crosstalk in tumour progression. Nat Rev Cancer (2021) 21:526–36. doi: 10.1038/s41568-021-00366-w PMC8740903 34103704

[187] Majeti R Chao MP Alizadeh AA Pang WW Jaiswal S Gibbs KD. CD47 is an adverse prognostic factor and therapeutic antibody target on human acute myeloid leukemia stem cells. Cell (2009) 138:286–99. doi: 10.1016/j.cell.2009.05.045 PMC2726837 19632179

[188] Zhang Y Sime W Juhas M Sjölander A. Crosstalk between colon cancer cells and macrophages *via* inflammatory mediators and CD47 promotes tumour cell migration. Eur J Cancer Oxf Engl 1990 (2013) 49:3320–34. doi: 10.1016/j.ejca.2013.06.005 23810249

[189] Guan B Li H Yao J Guo J Yu F Li G. CCL3-CCR5 axis promotes cell migration and invasion of colon adenocarcinoma *via* Akt signaling pathway. Environ Toxicol (2023) 38:172–84. doi: 10.1002/tox.23675 36346222

[190] Novak M Koprivnikar Krajnc M Hrastar B Breznik B Majc B Mlinar M. CCR5-mediated signaling is involved in invasion of glioblastoma cells in its microenvironment. Int J Mol Sci (2020) 21:4199. doi: 10.3390/ijms21124199 32545571 PMC7352708

[191] Yan J Zhao Q Wang J Tian X Wang J Xia X. FGL2-wired macrophages secrete CXCL7 to regulate the stem-like functionality of glioma cells. Cancer Lett (2021) 506:83–94. doi: 10.1016/j.canlet.2021.02.021 33676940 PMC8009861

[192] Radharani NNV Yadav AS Nimma R Kumar TVS Bulbule A Chanukuppa V. Tumor-associated macrophage derived IL-6 enriches cancer stem cell population and promotes breast tumor progression *via* Stat-3 pathway. Cancer Cell Int (2022) 22:122. doi: 10.1186/s12935-022-02527-9 35300689 PMC8932105

[193] Yang J Liao D Chen C Liu Y Chuang T-H Xiang R. Tumor-associated macrophages regulate murine breast cancer stem cells through a novel paracrine EGFR/stat3/sox-2 signaling pathway. Stem Cells (2013) 31:248–58. doi: 10.1002/stem.1281 23169551

[194] Jinushi M Chiba S Yoshiyama H Masutomi K Kinoshita I Dosaka-Akita H. Tumor-associated macrophages regulate tumorigenicity and anticancer drug responses of cancer stem/initiating cells. Proc Natl Acad Sci U.S.A. (2011) 108:12425–30. doi: 10.1073/pnas.1106645108 PMC3145680 21746895

[195] Chang J Li H Zhu Z Mei P Hu W Xiong X. microRNA-21-5p from M2 macrophage-derived extracellular vesicles promotes the differentiation and activity of pancreatic cancer stem cells by mediating KLF3. Cell Biol Toxicol (2022) 38:577–90. doi: 10.1007/s10565-021-09597-x PMC9343318 33728488

[196] Okuda H Kobayashi A Xia B Watabe M Pai SK Hirota S. Hyaluronan synthase HAS2 promotes tumor progression in bone by stimulating the interaction of breast cancer stem–like cells with macrophages and stromal cells. Cancer Res (2012) 72:537–47. doi: 10.1158/0008-5472.CAN-11-1678 PMC3404816 22113945

[197] Liu D Lu Q Wang X Wang J Lu N Jiang Z. LSECtin on tumor-associated macrophages enhances breast cancer stemness *via* interaction with its receptor BTN3A3. Cell Res (2019) 29:365–78. doi: 10.1038/s41422-019-0155-6 PMC6796923 30858559

[198] Zang X Zhang X Hu H Qiao M Zhao X Deng Y. Targeted delivery of zoledronate to tumor-associated macrophages for cancer immunotherapy. Mol Pharm (2019) 16:2249–58. doi: 10.1021/acs.molpharmaceut.9b00261 30969779

[199] Colon-Echevarria CB Ortiz T Maldonado L Hidalgo-Vargas MJ Pérez-Morales J Aquino-Acevedo AN. Zoledronic acid abrogates restraint stress-induced macrophage infiltration, PDGF-AA expression, and ovarian cancer growth. Cancers (2020) 12:2671. doi: 10.3390/cancers12092671 32962103 PMC7563308

[200] Hiroshima Y Maawy A Hassanein MK Menen R Momiyama M Murakami T. The tumor-educated-macrophage increase of Malignancy of human pancreatic cancer is prevented by zoledronic acid. PloS One (2014) 9:e103382. doi: 10.1371/journal.pone.0103382 25116261 PMC4130525

[201] Rogers TL Wind N Hughes R Nutter F Brown HK Vasiliadou I. Macrophages as potential targets for zoledronic acid outside the skeleton—evidence from in *vitro* and in *vivo* models. Cell Oncol (2013) 36:505–14. doi: 10.1007/s13402-013-0156-2 PMC13007476 24177992

[202] Boimel PJ Smirnova T Zhou ZN Wyckoff J Park H Coniglio SJ. Contribution of CXCL12 secretion to invasion of breast cancer cells. Breast Cancer Res BCR (2012) 14:R23. doi: 10.1186/bcr3108 22314082 PMC3496141

[203] Shih C-T Shiau C-W Chen Y-L Chen L-J Chao T-I Wang C-Y. TD-92, a novel erlotinib derivative, depletes tumor-associated macrophages in non-small cell lung cancer *via* down-regulation of CSF-1R and enhances the anti-tumor effects of anti-PD-1. Cancer Lett (2021) 498:142–51. doi: 10.1016/j.canlet.2020.10.043 33232786

[204] Kuemmel S Campone M Loirat D Lopez RL Beck JT De Laurentiis M. A randomized phase II study of anti-CSF1 monoclonal antibody lacnotuzumab (MCS110) combined with gemcitabine and carboplatin in advanced triple-negative breast cancer. Clin Cancer Res (2022) 28:106–15. doi: 10.1158/1078-0432.CCR-20-3955 34615719

[205] Dowlati A Harvey RD Carvajal RD Hamid O Klempner SJ Kauh JSW. LY3022855, an anti–colony stimulating factor-1 receptor (CSF-1R) monoclonal antibody, in patients with advanced solid tumors refractory to standard therapy: phase 1 dose-escalation trial. Invest New Drugs (2021) 39:1057–71. doi: 10.1007/s10637-021-01084-8 33624233

[206] Xu J Escamilla J Mok S David J Priceman S West B. CSF1R signaling blockade stanches tumor-infiltrating myeloid cells and improves the efficacy of radiotherapy. Cancer Res (2013) 73:2782–94. doi: 10.1158/0008-5472.CAN-12-3981 PMC4097014 23418320

[207] MacDonald KPA Palmer JS Cronau S Seppanen E Olver S Raffelt NC. An antibody against the colony-stimulating factor 1 receptor depletes the resident subset of monocytes and tissue- and tumor-associated macrophages but does not inhibit inflammation. Blood (2010) 116:3955–63. doi: 10.1182/blood-2010-02-266296 20682855

[208] Li X Yao W Yuan Y Chen P Li B Li J. Targeting of tumour-infiltrating macrophages *via* CCL2/CCR2 signalling as a therapeutic strategy against hepatocellular carcinoma. Gut (2017) 66:157–67. doi: 10.1136/gutjnl-2015-310514 26452628

[209] Cho HR Kumari N Thi Vu H Kim H Park C-K Choi SH. Increased antiangiogenic effect by blocking CCL2-dependent macrophages in a rodent glioblastoma model: correlation study with dynamic susceptibility contrast perfusion MRI. Sci Rep (2019) 9:11085. doi: 10.1038/s41598-019-47438-4 31366997 PMC6668454

[210] Nywening TM Wang-Gillam A Sanford DE Belt BA Panni RZ Cusworth BM. Targeting tumour-associated macrophages with CCR2 inhibition in combination with FOLFIRINOX in patients with borderline resectable and locally advanced pancreatic cancer: a single-centre, open-label, dose-finding, non-randomised, phase 1b trial. Lancet Oncol (2016) 17:651–62. doi: 10.1016/S1470-2045(16)00078-4 PMC5407285 27055731

[211] Linehan D Noel MS Hezel AF Wang-Gillam A Eskens F Sleijfer S. Overall survival in a trial of orally administered CCR2 inhibitor CCX872 in locally advanced/metastatic pancreatic cancer: Correlation with blood monocyte counts. J Clin Oncol (2018) 36:92–2. doi: 10.1200/JCO.2018.36.5_suppl.92

[212] Petty AJ Owen DH Yang Y Huang X. Targeting tumor-associated macrophages in cancer immunotherapy. Cancers (2021) 13:5318. doi: 10.3390/cancers13215318 34771482 PMC8582510

[213] Brana I Calles A LoRusso PM Yee LK Puchalski TA Seetharam S. Carlumab, an anti-C-C chemokine ligand 2 monoclonal antibody, in combination with four chemotherapy regimens for the treatment of patients with solid tumors: an open-label, multicenter phase 1b study. Target Oncol (2015) 10:111–23. doi: 10.1007/s11523-014-0320-2 24928772

[214] Ban Y Mai J Li X Mitchell-Flack M Zhang T Zhang L. Targeting autocrine CCL5-CCR5 axis reprograms immunosuppressive myeloid cells and reinvigorates antitumor immunity. Cancer Res (2017) 77:2857–68. doi: 10.1158/0008-5472.CAN-16-2913 PMC5484057 28416485

[215] Zhang M Huang L Ding G Huang H Cao G Sun X. Interferon gamma inhibits CXCL8-CXCR2 axis mediated tumor-associated macrophages tumor trafficking and enhances anti-PD1 efficacy in pancreatic cancer. J Immunother Cancer (2020) 8:e000308. doi: 10.1136/jitc-2019-000308 32051287 PMC7057481

[216] Jung K Heishi T Incio J Huang Y Beech EY Pinter M. Targeting CXCR4-dependent immunosuppressive Ly6Clow monocytes improves antiangiogenic therapy in colorectal cancer. Proc Natl Acad Sci U.S.A. (2017) 114:10455–60. doi: 10.1073/pnas.1710754114 PMC5625928 28900008

[217] O’Hara MH Messersmith W Kindler H Zhang W Pitou C Szpurka AM. Safety and pharmacokinetics of CXCR4 peptide antagonist, LY2510924, in combination with durvalumab in advanced refractory solid tumors. J Pancreat Cancer (2020) 6:21–31. doi: 10.1089/pancan.2019.0018 32219196 PMC7097682

[218] Bockorny B Macarulla T Semenisty V Borazanci E Feliu J Ponz-Sarvise M. Motixafortide and pembrolizumab combined to nanoliposomal irinotecan, fluorouracil, and folinic acid in metastatic pancreatic cancer: the COMBAT/KEYNOTE-202 trial. Clin Cancer Res (2021) 27:5020–7. doi: 10.1158/1078-0432.CCR-21-0929 34253578

[219] Wesolowski R Sharma N Reebel L Rodal MB Peck A West BL. Phase Ib study of the combination of pexidartinib (PLX3397), a CSF-1R inhibitor, and paclitaxel in patients with advanced solid tumors. Ther Adv Med Oncol (2019) 11:1758835919854238. doi: 10.1177/1758835919854238 31258629 PMC6589951

[220] Papadavid E Stratigos AJ Falagas ME. Imiquimod: an immune response modifier in the treatment of precancerous skin lesions and skin cancer. Expert Opin Pharmacother (2007) 8:1743–55. doi: 10.1517/14656566.8.11.1743 17685890

[221] Adams S Kozhaya L Martiniuk F Meng T-C Chiriboga L Liebes L. Topical TLR7 agonist imiquimod can induce immune-mediated rejection of skin metastases in patients with breast cancer. Clin Cancer Res Off J Am Assoc Cancer Res (2012) 18:6748–57. doi: 10.1158/1078-0432.CCR-12-1149 PMC3580198 22767669

[222] Zanganeh S Hutter G Spitler R Lenkov O Mahmoudi M Shaw A. Iron oxide nanoparticles inhibit tumour growth by inducing pro-inflammatory macrophage polarization in tumour tissues. Nat Nanotechnol (2016) 11:986–94. doi: 10.1038/nnano.2016.168 PMC5198777 27668795

[223] Kapp K Volz B Oswald D Wittig B Baumann M Schmidt M. Beneficial modulation of the tumor microenvironment and generation of anti-tumor responses by TLR9 agonist lefitolimod alone and in combination with checkpoint inhibitors. OncoImmunology (2019) 8:e1659096. doi: 10.1080/2162402X.2019.1659096 31741757 PMC6844329

[224] Thomas M Ponce-Aix S Navarro A Riera-Knorrenschild J Schmidt M Wiegert E. Immunotherapeutic maintenance treatment with toll-like receptor 9 agonist lefitolimod in patients with extensive-stage small-cell lung cancer: results from the exploratory, controlled, randomized, international phase II IMPULSE study. Ann Oncol (2018) 29:2076–84. doi: 10.1093/annonc/mdy326 PMC6225892 30137193

[225] Rolfo C Giovannetti E Martinez P McCue S Naing A. Applications and clinical trial landscape using Toll-like receptor agonists to reduce the toll of cancer. NPJ Precis Oncol (2023) 7:1–11. doi: 10.1038/s41698-023-00364-1 36890302 PMC9995514

[226] Long KB Gladney WL Tooker GM Graham K Fraietta JA Beatty GL. IFNγ and CCL2 cooperate to redirect tumor-infiltrating monocytes to degrade fibrosis and enhance chemotherapy efficacy in pancreatic carcinoma. Cancer Discov (2016) 6:400–13. doi: 10.1158/2159-8290.CD-15-1032 PMC4843521 26896096

[227] O’Hara MH O’Reilly EM Varadhachary G Wolff RA Wainberg ZA Ko AH. CD40 agonistic monoclonal antibody APX005M (sotigalimab) and chemotherapy, with or without nivolumab, for the treatment of metastatic pancreatic adenocarcinoma: an open-label, multicentre, phase 1b study. Lancet Oncol (2021) 22:118–31. doi: 10.1016/S1470-2045(20)30532-5 33387490

[228] Moradi-Chaleshtori M Bandehpour M Soudi S Mohammadi-Yeganeh S Hashemi SM. *In vitro* and in *vivo* evaluation of anti-tumoral effect of M1 phenotype induction in macrophages by miR-130 and miR-33 containing exosomes. Cancer Immunol Immunother (2021) 70:1323–39. doi: 10.1007/s00262-020-02762-x PMC10991174 33140190

[229] Cabrales P. RRx-001 acts as a dual small molecule checkpoint inhibitor by downregulating CD47 on cancer cells and SIRP-α on monocytes/macrophages. Transl Oncol (2019) 12:626–32. doi: 10.1016/j.tranon.2018.12.001 PMC6370857 30738349

[230] Oronsky B Paulmurugan R Foygel K Scicinski J Knox SJ Peehl D. RRx-001: a systemically non-toxic M2-to-M1 macrophage stimulating and prosensitizing agent in Phase II clinical trials. Expert Opin Investig Drugs (2017) 26:109–19. doi: 10.1080/13543784.2017.1268600 27935336

[231] Advani R Flinn I Popplewell L Forero A Bartlett NL Ghosh N. CD47 blockade by hu5F9-G4 and rituximab in non-hodgkin’s lymphoma. N Engl J Med (2018) 379:1711–21. doi: 10.1056/NEJMoa1807315 PMC8058634 30380386

[232] Veillette A Tang Z. Signaling regulatory protein (SIRP)α-CD47 blockade joins the ranks of immune checkpoint inhibition. J Clin Oncol (2019) 37:1012–4. doi: 10.1200/JCO.19.00121 30811295

[233] Upton R Banuelos A Feng D Biswas T Kao K McKenna K. Combining CD47 blockade with trastuzumab eliminates HER2-positive breast cancer cells and overcomes trastuzumab tolerance. Proc Natl Acad Sci (2021) 118:e2026849118. doi: 10.1073/pnas.2026849118 34257155 PMC8307693

[234] Wang H Sun Y Zhou X Chen C Jiao L Li W. CD47/SIRPα blocking peptide identification and synergistic effect with irradiation for cancer immunotherapy. J Immunother Cancer (2020) 8:e000905. doi: 10.1136/jitc-2020-000905 33020240 PMC7537338

[235] Xi Q Zhang J Yang G Zhang L Chen Y Wang C. Restoration of miR-340 controls pancreatic cancer cell CD47 expression to promote macrophage phagocytosis and enhance antitumor immunity. J Immunother Cancer (2020) 8:e000253. doi: 10.1136/jitc-2019-000253 32503944 PMC7279671

[236] Kuo TC Chen A Harrabi O Sockolosky JT Zhang A Sangalang E. Targeting the myeloid checkpoint receptor SIRPα potentiates innate and adaptive immune responses to promote anti-tumor activity. J Hematol OncolJ Hematol Oncol (2020) 13:160. doi: 10.1186/s13045-020-00989-w 33256806 PMC7706287

[237] Dodagatta-Marri E Meyer DS Reeves MQ Paniagua R To MD Binnewies M. α-PD-1 therapy elevates Treg/Th balance and increases tumor cell pSmad3 that are both targeted by α-TGFβ antibody to promote durable rejection and immunity in squamous cell carcinomas. J Immunother Cancer (2019) 7:62. doi: 10.1186/s40425-018-0493-9 30832732 PMC6399967

[238] Squibb B-M. A phase 1/2 study of relatlimab (Anti-LAG-3 monoclonal antibody) administered in combination with both nivolumab (Anti-PD-1 monoclonal antibody) and BMS-986205 (IDO1 inhibitor) or in combination with both nivolumab and ipilimumab (Anti-CTLA-4 monoclonal antibody) in advanced Malignant tumors (2023). clinicaltrials.gov. Available at: https://clinicaltrials.gov/study/NCT03459222 (Accessed January 1, 2023).

[239] La Fleur L Botling J He F Pelicano C Zhou C He C. Targeting MARCO and IL37R on immunosuppressive macrophages in lung cancer blocks regulatory T cells and supports cytotoxic lymphocyte function. Cancer Res (2021) 81:956–67. doi: 10.1158/0008-5472.CAN-20-1885 33293426

[240] He Y Pei J-H Li X-Q Chi G. IL-35 promotes EMT through STAT3 activation and induces MET by promoting M2 macrophage polarization in HCC. Biochem Biophys Res Commun (2021) 559:35–41. doi: 10.1016/j.bbrc.2021.04.050 33932898

[241] Moyes KW Lieberman NAP Kreuser SA Chinn H Winter C Deutsch G. Genetically engineered macrophages: A potential platform for cancer immunotherapy. Hum Gene Ther (2017) 28:200–15. doi: 10.1089/hum.2016.060 27758144

[242] Morrissey MA Williamson AP Steinbach AM Roberts EW Kern N Headley MB. Chimeric antigen receptors that trigger phagocytosis. eLife (2018) 7:e36688. doi: 10.7554/eLife.36688 29862966 PMC6008046

[243] Carisma Therapeutics Inc. A phase 1, first in human study of adenovirally transduced autologous macrophages engineered to contain an anti-HER2 chimeric antigen receptor in subjects with HER2 overexpressing solid tumors (2022). clinicaltrials.gov. Available at: https://clinicaltrials.gov/study/NCT04660929 (Accessed January 1, 2023).

[244] Kalbasi A Komar C Tooker GM Liu M Lee JW Gladney WL. Tumor-derived CCL2 mediates resistance to radiotherapy in pancreatic ductal adenocarcinoma. Clin Cancer Res Off J Am Assoc Cancer Res (2017) 23:137–48. doi: 10.1158/1078-0432.CCR-16-0870 PMC5195913 27354473

[245] Wang D Yue DL Wang D Chen XF Yin XY Wang YP. [Aspirin inhibits cell stemness of esophageal cancer by downregulation of chemokine CCL2]. Zhonghua Zhong Liu Za Zhi (2018) 40:744–9. doi: 10.3760/cma.j.issn.0253-3766.2018.10.005 30392338

[246] Taromi S Kayser G Catusse J von Elverfeldt D Reichardt W Braun F. CXCR4 antagonists suppress small cell lung cancer progression. Oncotarget (2016) 7:85185–95. doi: 10.18632/oncotarget.13238 PMC5356728 27835905

[247] Huelse JM Fridlyand DM Earp S DeRyckere D Graham DK. MERTK in cancer therapy: Targeting the receptor tyrosine kinase in tumor cells and the immune system. Pharmacol Ther (2020) 213:107577. doi: 10.1016/j.pharmthera.2020.107577 32417270 PMC9847360

[248] Lahey KC Gadiyar V Hill A Desind S Wang Z Davra V. Mertk: An emerging target in cancer biology and immuno-oncology. Int Rev Cell Mol Biol (2022) 368:35–59. doi: 10.1016/bs.ircmb.2022.04.004 35636929 PMC9994207

[249] Myers KV Amend SR Pienta KJ. Targeting Tyro3, Axl and MerTK (TAM receptors): implications for macrophages in the tumor microenvironment. Mol Cancer (2019) 18:94. doi: 10.1186/s12943-019-1022-2 31088471 PMC6515593

[250] Chen C-J Liu Y-P. MERTK inhibition: potential as a treatment strategy in EGFR tyrosine kinase inhibitor-resistant non-small cell lung cancer. Pharmaceuticals (2021) 14:130. doi: 10.3390/ph14020130 33562150 PMC7915726

[251] Cummings CT Zhang W Davies KD Kirkpatrick GD Zhang D DeRyckere D. Small molecule inhibition of MERTK is efficacious in non–small cell lung cancer models independent of driver oncogene status. Mol Cancer Ther (2015) 14:2014–22. doi: 10.1158/1535-7163.MCT-15-0116 PMC4704683 26162689

[252] DeRyckere D Lee-Sherick AB Huey MG Hill AA Tyner JW Jacobsen KM. UNC2025, a MERTK small-molecule inhibitor, is therapeutically effective alone and in combination with methotrexate in leukemia models. Clin Cancer Res (2017) 23:1481–92. doi: 10.1158/1078-0432.CCR-16-1330 PMC5354980 27649555

[253] Gao L Yu S Zhang X. Hypothesis: Tim-3/galectin-9, a new pathway for leukemia stem cells survival by promoting expansion of myeloid-derived suppressor cells and differentiating into tumor-associated macrophages. Cell Biochem Biophys (2014) 70:273–7. doi: 10.1007/s12013-014-9900-0 24639110

[254] Sarkar S Döring A Zemp FJ Silva C Lun X Wang X. Therapeutic activation of macrophages and microglia to suppress brain tumor-initiating cells. Nat Neurosci (2014) 17:46–55. doi: 10.1038/nn.3597 24316889

[255] Brown JR Chan DK Shank JJ Griffith KA Fan H Szulawski R. Phase II clinical trial of metformin as a cancer stem cell-targeting agent in ovarian cancer. JCI Insight (2020) 5:e133247. doi: 10.1172/jci.insight.133247 32369446 PMC7308054

[256] Fischer MM Cancilla B Yeung VP Cattaruzza F Chartier C Murriel CL. WNT antagonists exhibit unique combinatorial antitumor activity with taxanes by potentiating mitotic cell death. Sci Adv (2017) 3:e1700090. doi: 10.1126/sciadv.1700090 28691093 PMC5479655

[257] Ernst E. The role of complementary and alternative medicine in cancer. Lancet Oncol (2000) 1:176–80. doi: 10.1016/S1470-2045(00)00031-0 11905656

[258] Waldman AD Fritz JM Lenardo MJ. A guide to cancer immunotherapy: from T cell basic science to clinical practice. Nat Rev Immunol (2020) 20:651–68. doi: 10.1038/s41577-020-0306-5 PMC7238960 32433532

[259] Deng L-J Qi M Li N Lei Y-H Zhang D-M Chen J-X. Natural products and their derivatives: Promising modulators of tumor immunotherapy. J Leukoc Biol (2020) 108:493–508. doi: 10.1002/JLB.3MR0320-444R 32678943 PMC7496826

[260] Tripathi S Bruch D Kittur DS. Ginger extract inhibits LPS induced macrophage activation and function. BMC Complement Altern Med (2008) 8:1. doi: 10.1186/1472-6882-8-1 18173849 PMC2234390

[261] Wyburn KR Jose MD Wu H Atkins RC Chadban SJ. The role of macrophages in allograft rejection. Transplantation (2005) 80:1641–7. doi: 10.1097/01.tp.0000173903.26886.20 16378052

[262] Kim HW Murakami A Abe M Ozawa Y Morimitsu Y Williams MV. Suppressive effects of mioga ginger and ginger constituents on reactive oxygen and nitrogen species generation, and the expression of inducible pro-inflammatory genes in macrophages. Antioxid Redox Signal (2005) 7:1621–9. doi: 10.1089/ars.2005.7.1621 16356125

[263] Cao M Yan H Han X Weng L Wei Q Sun X. Ginseng-derived nanoparticles alter macrophage polarization to inhibit melanoma growth. J Immunother Cancer (2019) 7:326. doi: 10.1186/s40425-019-0817-4 31775862 PMC6882204

[264] Hossain DMS Bhattacharyya S Das T Sa G. Curcumin: the multi-targeted therapy for cancer regression. Front Biosci Sch Ed (2012) 4:335–55. doi: 10.2741/272 22202064

[265] Paul S Sa G. Curcumin as an adjuvant to cancer immunotherapy. Front Oncol (2021) 11:675923. doi: 10.3389/fonc.2021.675923 34485117 PMC8415504

[266] Bhattacharyya S Md Sakib Hossain D Mohanty S Sankar Sen G Chattopadhyay S Banerjee S. Curcumin reverses T cell-mediated adaptive immune dysfunctions in tumor-bearing hosts. Cell Mol Immunol (2010) 7:306–15. doi: 10.1038/cmi.2010.11 PMC4003225 20305684

[267] Sa G Das T. Anti cancer effects of curcumin: cycle of life and death. Cell Div (2008) 3:14. doi: 10.1186/1747-1028-3-14 18834508 PMC2572158

[268] Bhandarkar SS Arbiser JL. CURCUMIN AS AN INHIBITOR OF ANGIOGENESIS. In: Aggarwal BB Surh Y-J Shishodia S, editors. The molecular targets and therapeutic uses of curcumin in health and disease. ADVANCES IN EXPERIMENTAL MEDICINE AND BIOLOGY. Boston, MA: Springer US (2007). p. 185–95. doi: 10.1007/978-0-387-46401-5_7 17569211

[269] Davoodvandi A Farshadi M Zare N Akhlagh SA Alipour Nosrani E Mahjoubin-Tehran M. Antimetastatic effects of curcumin in oral and gastrointestinal cancers. Front Pharmacol (2021) 12:668567. doi: 10.3389/fphar.2021.668567 34456716 PMC8386020

[270] Bhattacharyya S Mandal D Saha B Sen GS Das T Sa G. Curcumin prevents tumor-induced T cell apoptosis through Stat-5a-mediated Bcl-2 induction. J Biol Chem (2007) 282:15954–64. doi: 10.1074/jbc.M608189200 17392282

[271] Jakubczyk K Drużga A Katarzyna J Skonieczna-Żydecka K. Antioxidant potential of curcumin-A meta-analysis of randomized clinical trials. Antioxid Basel Switz (2020) 9:1092. doi: 10.3390/antiox9111092 PMC7694612 33172016

[272] Mukherjee S Mazumdar M Chakraborty S Manna A Saha S Khan P. Curcumin inhibits breast cancer stem cell migration by amplifying the E-cadherin/β-catenin negative feedback loop. Stem Cell Res Ther (2014) 5:116. doi: 10.1186/scrt506 25315241 PMC4445824

[273] Dos Santos AP Cardoso TN Waisse S Bonamin LV. Homeopathy in experimental cancer models: A systematic review. Homeopathy J Fac Homeopathy (2021) 110:76–85. doi: 10.1055/s-0040-1716369 33348419

[274] Yadav R Jee B Rao KS. How homeopathic medicine works in cancer treatment: deep insight from clinical to experimental studies. J Exp Ther Oncol (2019) 13:71–6. 30658031

[275] Fuselier C Quemener S Dufay E Bour C Boulagnon-Rombi C Bouland N. Anti-tumoral and anti-angiogenic effects of low-diluted phenacetinum on melanoma. Front Oncol (2021) 11:597503. doi: 10.3389/fonc.2021.597503 33747916 PMC7966719

[276] Gonçalves JP Dos Santos MLF Rossi GR Costa Gagosian VS de Oliveira CC. Differential effects of Zincum metallicum on cell models. Homeopathy J Fac Homeopathy (2017) 106:171–80. doi: 10.1016/j.homp.2017.02.004 28844290

[277] Seligmann IC Lima PDL Cardoso PCS Khayat AS Bahia MO Buchi D de F. The anticancer homeopathic composite “Canova Method” is not genotoxic for human lymphocytes in *vitro* . Genet Mol Res GMR (2003) 2:223–8. 14966688

[278] Ghosh AK. A short history of the development of homeopathy in India. Homeopathy J Fac Homeopathy (2010) 99:130–6. doi: 10.1016/j.homp.2009.10.001 20471616

[279] Saha S Hossain DMS Mukherjee S Mohanty S Mazumdar M Mukherjee S. Calcarea carbonica induces apoptosis in cancer cells in p53-dependent manner *via* an immuno-modulatory circuit. BMC Complement Altern Med (2013) 13:230. doi: 10.1186/1472-6882-13-230 24053127 PMC3856502

[280] Xiang X Wang J Lu D Xu X. Targeting tumor-associated macrophages to synergize tumor immunotherapy. Signal Transduct Target Ther (2021) 6:1–12. doi: 10.1038/s41392-021-00484-9 33619259 PMC7900181

[281] Liu C-Q Xu J Zhou Z-G Jin L-L Yu X-J Xiao G. Expression patterns of programmed death ligand 1 correlate with different microenvironments and patient prognosis in hepatocellular carcinoma. Br J Cancer (2018) 119:80–8. doi: 10.1038/s41416-018-0144-4 PMC6035200 29921949

[282] Xu S Wang C Yang L Wu J Li M Xiao P. Targeting immune checkpoints on tumor-associated macrophages in tumor immunotherapy. Front Immunol (2023) 14:1199631. doi: 10.3389/fimmu.2023.1199631 37313405 PMC10258331

[283] Mariathasan S Turley SJ Nickles D Castiglioni A Yuen K Wang Y. TGFβ attenuates tumour response to PD-L1 blockade by contributing to exclusion of T cells. Nature (2018) 554:544–8. doi: 10.1038/nature25501 PMC6028240 29443960

[284] Schmid P Adams S Rugo HS Schneeweiss A Barrios CH Iwata H. Atezolizumab and nab-paclitaxel in advanced triple-negative breast cancer. N Engl J Med (2018) 379:2108–21. doi: 10.1056/NEJMoa1809615 30345906

